# Diet Quality Scores, Obesity and Metabolic Syndrome in Children and Adolescents: A Systematic Review and Meta-Analysis

**DOI:** 10.1007/s13679-024-00589-6

**Published:** 2024-09-27

**Authors:** Alicia Larruy-García, Lubna Mahmood, María L. Miguel-Berges, Guiomar Masip, Miguel Seral-Cortés, Pilar De Miguel-Etayo, Luis A. Moreno

**Affiliations:** 1 Growth, Exercise, Nutrition and Development (GENUD) Research Group, Instituto Agroalimentario de Aragón (IA2), Physiatry and Nursing Department, Faculty of Health Sciences, Instituto de Investigación Sanitaria de Aragón (IIS Aragón), Universidad de Zaragoza, Pedro Cerbuna Street, 50009 Zaragoza, Spain; 2 CIBER. Fisiopatología de La Obesidad y Nutrición (CIBEROBN), Instituto de Salud Carlos III (ISCIII), Madrid, Spain

**Keywords:** Diet quality, Mediterranean diet, Obesity, Metabolic syndrome, Meta-analysis, Systematic review

## Abstract

**Purpose of Review:**

We aimed to examine the relationship between various diet quality scores and obesity and Metabolic Syndrome (MetS) in children and adolescents.

**Recent Findings:**

Obesity and MetS, which increase the risk of type 2 diabetes and cardiovascular disease from childhood through adolescence, have been associated with adherence to various diet quality scores.

**Summary:**

A systematic search was performed in PubMed/Medline, Scopus, SciELO, Embase, and Cochrane, covering the period until March 2024. Two researchers evaluated 3,519 studies according to the inclusion criteria. Finally, 73 articles that analysed the relationship between diet quality scores and obesity and MetS were included, and 6 of them were included in a meta-analysis. Children younger than 12 years old showed statistically significant differences indicating a higher Mediterranean diet (MD) score adherence compared to those with a low score adherence for BMI (MD = 0.33 kg/m^2^, 95% CI: 0.01, 0.64) and WC values (MD = 1.21 cm, 95% CI: 0.50, 1.93). Additionally, in the meta-regression analysis, boys showed stronger associations for BMI, z-score BMI and WC (β = 19.82, 95% CI: 17.62, 22.03, β = 0.64, 95% CI: 0.33, 0.96 and β = 67.03, 95% CI: 57.29, 76.77, respectively). Studies in this review suggest an association between high adherence to different diet quality scores and low BMI. Meta-analysis assessing the association between adherence to the MD and BMI, and WC, showed a protective effect of the MD pattern against obesity outcomes. This systematic review and meta-analyses provided evidence on the effect of the diet quality on obesity and MetS in children and adolescents.

**Supplementary Information:**

The online version contains supplementary material available at 10.1007/s13679-024-00589-6.

## Introduction

Obesity has become a major global epidemic with substantial health implications for children and adolescents worldwide [1, 2]. Weight problems and obesity are increasing in most of the EU Member States [3]. Overall, in the 33 countries of the WHO European region that collected data in the fifth round of the Childhood Obesity Surveillance Initiative (COSI), 29% of children aged 7–9 years old were affected by having overweight and/or obesity. The prevalence ranged from 6% in Tajikistan to 43% in Cyprus (3). In addition, obesity among children and adolescents tends to persist into later life, thus increasing the risk of obesity during adulthood [3, 4].

It is well known that eating habits and dietary patterns acquired during childhood are likely to be maintained into adulthood [5]. Obesity is considered as a risk factor in the development of Metabolic Syndrome (MetS) [2, 4, 5]. MetS is a complex disorder affecting individuals across all age groups, including children and adolescents [1, 2]. Having MetS increases the risk of developing type 2 diabetes mellitus and cardiovascular disease [6]. In 2020, the worldwide prevalence of MetS was estimated to be 3% in children and 5% in adolescents [7]*.*

The concept of diet quality has recently gained considerable attention in nutritional research. Despite its widespread use, it is often poorly defined and remains difficult to measure [8]. Diet quality describes the individual´s compliance to dietary recommendations that are often reflected in food-based dietary guidelines [8]. Moreover, in children, it can also refer to both the amount of nutrients and the uptake of specific nutrients from foods to support growth and maintenance [9]. Hence, a high diet quality reflects improved food intake [10].

Dietary Quality Scores or Indices (DQSs) are tools aiming to evaluate an individual’s overall diet and categorize individuals according to the extent to which their dietary habits are healthy [11]. The three major categories of DQSs are nutrients-based, food frequencies/food groups-based, or a combination of both [11]. Moreover, some DQSs are designed for specific countries or age groups and may not be applicable to other populations [12]. The DQI (Diet Quality Index), MDS (Mediterranean Diet Score), and HDI (Healthy Diet Index) are three internationally recognized and widely used DQSs [11–13].

Although low diet quality has been linked to obesity, the relationship remains unclear. The association between dietary components reflected in DQSs and the weight status of children and adolescents has not been extensively studied [14]. A cross-sectional study on children assessing three predefined DQSs found that higher scores in the DQI and HDI were significantly associated with lower weight status and waist circumference (WC), while no significant associations were observed with the MDS [13]. A cross-sectional study on diet quality and adiposity in children found that consumption of a low diet quality during early childhood is linked to obesity at the age of 6 years old [14]. Similarly, a longitudinal study assessing adherence to the MD in children found that high DQIs at 3 years old were associated with a lower risk of obesity, while adherence to the MD itself did not show any association [15]. Furthermore, an intervention program involving adolescents, which assessed the associations between adherence to the DQI and changes in body composition, found a significant association between higher DQI scores and improved z-score body mass index (BMI) and fat-free mass index (FFMI) [16].

Although only a few studies have documented the association between DQSs and MetS. A cross-sectional study showed a low prevalence of MetS in those adolescents with a low Healthy Eating Index (HEI). The study found that lower scores of HEI were associated with several adverse components of MetS (i.e., low high-density lipoprotein (HDL) concentrations, high triglycerides concentrations and high blood pressure levels) [17]. Similarly, another cross-sectional study of 4,450 US adolescents found that the prevalence of MetS decreased with higher adherence to the overall HEI [15]. Another study also showed that MetS prevalence was 16 times higher in adolescents with overweight [18]. A systematic review of DQSs, adiposity and MetS among children showed that improvements in diet quality could be associated with lower adiposity levels, and lower risk of developing MetS or its individual components [19]. Moreover, a cross-sectional study of 135 participants aged 6–17 years old in Turkey suggested that higher DQSs were associated with a lower proportion of children with insulin resistance [20], which is considered a common trigger of MetS [21]. Similarly, a longitudinal study in Australian children found that those adhering to the least healthy diet showed impaired functional cardiovascular phenotypes and an increased risk of MetS [22].

Given the current importance of obesity and MetS and the limited number of studies investigating the association between dietary quality and these disorders in children and adolescents, this systematic review and meta-analysis aims to analyse various diet quality scores and their association with obesity and MetS in children and adolescents.

## Materials and Methods

This systematic review was conducted following the Preferred Reporting Items for Systematic reviews and Meta-Analyses (PRISMA) recommendations [23]. This systematic review was preregistered on the international prospective register of systematic reviews (PROSPERO: CRD42021236079).

Two independent researchers (AL-G and LM) conducted a comprehensive search for scientific articles in the following electronic databases: Medline/PubMed (National Library of Medicine of the USA), Cochrane, Scopus (Elsevier), Embase (Elsevier) and SciELO Science Citation Index (Clarivate Analytics), without restrictions on period and location. The research was structured and organized according to the Population, Intervention, Comparison, Outcomes and Study type (PICOS) model. The population of interest or health problem (P) consisted of participants aged 2 to 20 years old; intervention (I) referred to any DQS related to obesity or MetS; comparison (C) did not involve a specific comparator; outcomes (O) included obesity and MetS; and the types of studies (S) included were cross-sectional, cohort studies and clinical trials.

### Search Strategy

The descriptors were selected from the Health Sciences Descriptors (DeCS) dictionary and Medical Subject Heading Terms (MeSH), considering their widespread use by the scientific community for indexing articles in the PubMed database. Their suitability for the other databases used in the search was also considered.

The keywords and MeSH terms included in the search strategy were: “diet quality”, “diet patterns”, “diet score”, “Healthy Eating Indices, “diet (quality) index”, “diet indices”, “Dietary Approaches To Stop Hypertension”Mesh, “DASH Diet”, “Mediterranean Diet”, “Metabolic Syndrome”MeSH Terms, “Insulin Resistance”MeSH Terms, “insulin sensitive”, “Obesity”MeSH Terms, “Pediatric Obesity”MeSH Terms, “Body Composition”MeSH Terms, “Skinfold Thickness”MeSH Terms, “Body fat distribution”MeSH Terms, “Adiposity”MeSH Terms, “Body weight”MeSH Terms, “Body weight and measures”, “Body Mass Index”MeSH Terms, “Body fat distribution”MeSH Terms, “Fat mass index”, “Quetelet Index”, “Waist circumference”MeSH Terms, “Waist-hip ratio”MeSH Terms, “Waist-height ratio”MeSH Terms, “child”MeSH Terms, “Child, Preschool*”MeSH Terms, “adolescent”MeSH Terms, “Youth”, “Teen”, “Young people”. The search strategies applied were included in the Supplementary Material (Table S1).

A total of 3,519 articles were retrieved, and their titles were checked for duplications and relevance to the research topic. Subsequently, the retrieved articles were screened using the inclusion and exclusion criteria to identify their eligibility.

### Eligibility Criteria

The review question was defined as follows: What is the association between diet quality scores (DQSs) and obesity and MetS in children and adolescents?

Thus, in the present report, articles were considered eligible if they met these inclusion criteria: (a) studies based on human subjects between 2 to 20 years old, (b) studies published in English and/or Spanish language, (c) studies using quantitative methods that examine DQSs and their association with body composition characteristics such as BMI, z-score BMI, WC, skinfolds and percentage of body fat or fat mass index, and metabolic syndrome indicators such as blood pressure, HDL-cholesterol, triglycerides and homeostasis model assessment (HOMA)-Index, in children and adolescents, (d) peer-reviewed observational and intervention studies (i.e., cross-sectional studies…).

### Exclusion Criteria

Exclusion criteria comprised: (1) studies that they did not meet the above-mentioned inclusion criteria, (2) studies conducted in infants (< 2 years old) and among adults (> 20 years old), (3) meta-analyses, systematic reviews, literature reviews, narrative reviews, letters to the editor, and conference abstracts, (4) studies with missing information, unclear data, or unavailable in full text or in languages other than English or Spanish.

### Risk of Bias

The methodological quality of the included studies was independently assessed by the reviewers (AL-G and LM) according to the Cochrane risk of bias guidelines. This assessment was conducted blindly, with the names of the authors and journals masked, to avoid potential bias and conflicts of interest.

### Data Extraction

Data extraction for the study eligibility process was performed using a specific form for the systematic review, prepared by the researchers using an Excel file. Both researchers (AL-G and LM) independently added the extracted data to the file, which was then compared to ensure accuracy. Finally, another researcher (PM-E) reviewed the extracted data for verification. The first step of the screening process involved selecting articles based on their titles. Both researchers (AL-G and LM) independently reviewed all the titles and then reached a consensus on whether to include them or not. The next step was abstract selection and eligibility assessment. Articles selected based on the abstracts screening underwent full text review, and only those meeting all eligibility criteria were included. In cases of disagreement between the researchers, a third researcher (PM-E) made the final decision.

### Collected Data

After the initial screening, the full text of the selected articles underwent a standardized review and data extraction process conducted by two researchers (AL-G and LM), under the supervision of a third researcher (PM-E). The following information was extracted: author names, publication year, study year, country where the research was conducted, study design, sample size (N), sex of the subjects split into female and male groups (N, %), age of participants, DQS used, measures of obesity, measures of MetS, study results, and quality control assessment.

### Quality Assessment

The quality of the included studies was independently assessed by two of the authors (AL-G and LM) using the following tools: 1) for cross-sectional studies, the BSA Medical Sociology Group quality evaluation tool [24]; 2) for cohort studies, the Newcastle–Ottawa Scale [25]; 3) for intervention studies, the National Heart, Lung, and Blood Institute quality assessment tool for controlled intervention studies [26]. The results of the quality assessment are presented in supplementary Table Q1. Quality was rate as high, moderate, low, or very low according to the Grading of Recommendations Assessment, Development and Evaluation (GRADE) criteria [27].

### Statistical Analysis

Articles using the same DQS and similar obesity outcomes were considered for meta-analysis. Only the Mediterranean diet (MD) scores were included in the meta-analysis, as they were the only ones that provided the necessary data to perform the analysis.

For continuous data (BMI, kg/m^2^ and WC, cm), the mean difference with 95% confidence intervals (95% CI) was calculated to compare individuals with low adherence to the MD score versus those with high adherence. DerSimonian and Laird estimators using random-effects models were applied for continuous data. Effect sizes were calculated for each outcome.

Sources of heterogeneity were investigated through subgroup analyses comparing results by sex (boys vs. girls) and age (< 12 years old vs. > 12 years old). All analyses were performed by AL-G and MM-B using Open Meta [Analyst] software.

The heterogeneity of the studies was tested using the I^2^ statistic, with values of 50% to 75% indicating high heterogeneity and values > 75% indicating very high heterogeneity. The associated *p*-value of the heterogeneity of the studies was also calculated, with a nonsignificant result indicating absence of heterogeneity.

To explore the influence of potential sources of heterogeneity on the high MD score adherence, a meta-regression analysis assuming a random-effects model was performed considering BMI, z-score BMI and WC as predictors, grouped by sex and age.

## Results

**Fig. 1:**
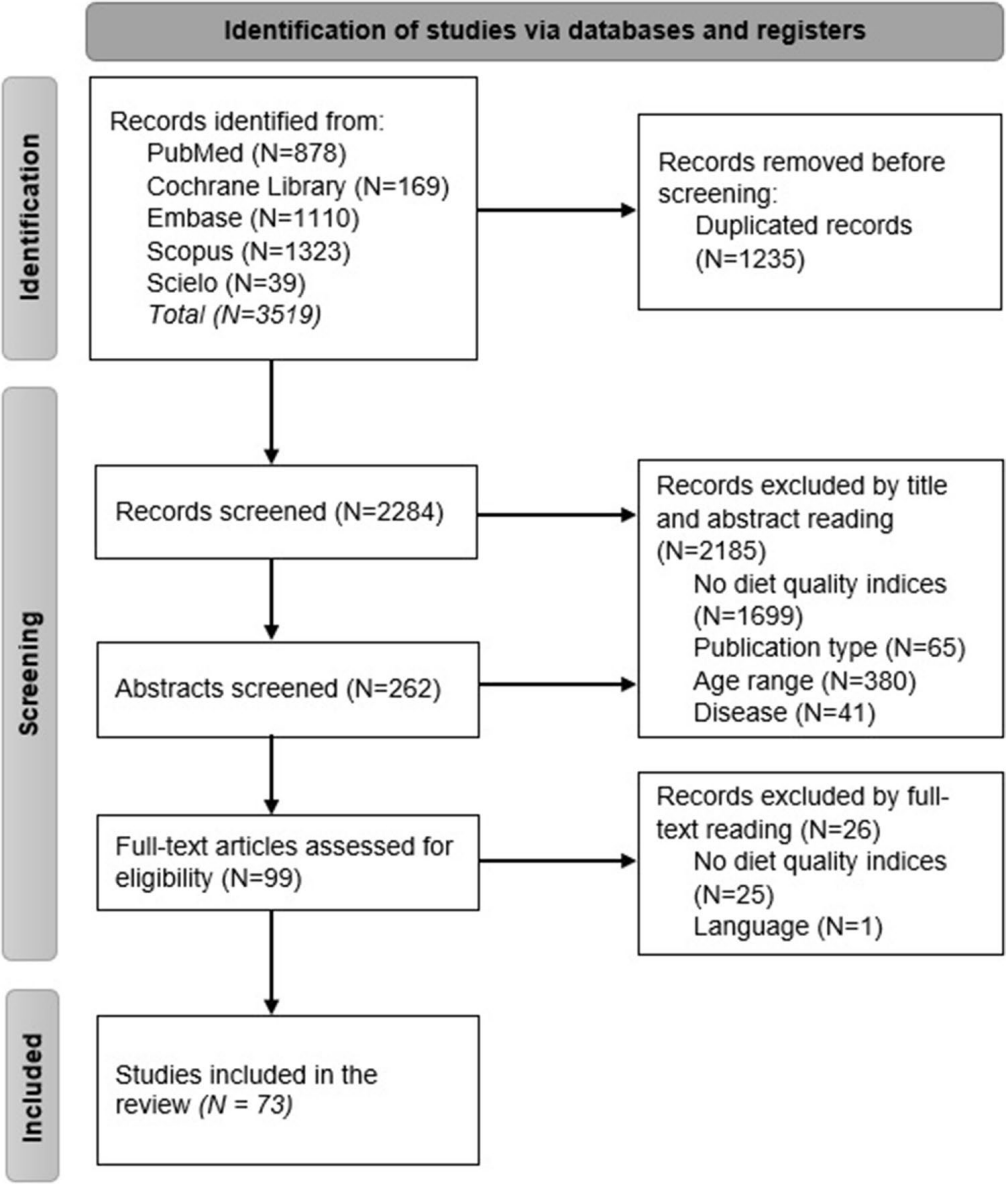
PRISMA flowchart of study selection

A flow chart summarizing the study selection procedure is presented in Fig. 1 The screening process involved searching five relevant electronic databases (PubMed, Cochrane, Scopus, Embase and SciELO), resulting in the retrieval of 3,519 articles. After removing duplicates, a total of 2,284 articles were screened. Following the initial screening based on article titles, 262 articles underwent abstract screening, and 99 articles were assessed based on full text to decide their final inclusion. The main reasons for study exclusion were the absence of diet quality scores (DQSs) (*n* = 1,604), inappropriate publication type (*n* = 59), unsuitable age range (*n* = 372), unrelated disease (*n* = 17) or non-English/Spanish language (*n* = 1). A total of 73 articles met the inclusion criteria and were included in this systematic review and meta-analysis.

Data extraction revealed 36 different DQSs assessing the association with obesity and/or MetS in children and adolescents, with various outcomes related to obesity and MetS indicators.

The characteristics of the selected studies are reported in Tables 1 and 2, divided by type of DQSs: “Mediterranean diet-based scores” and “different dietary quality scores”, respectively.

**Table 1:** Summary of the findings for different Mediterranean diet adherence indices and body composition and metabolic syndrome measures

Author and Publication Year	Recruitment Year; Country	Study Design	Population study (N); Boys (N,%)	Age	Mediterranean Diet Quality Index	Body fat Composition Measures	Metabolic Syndrome Measures	Summary of main results
Alonso F. J. et al. 2014	2011–2012; Spain	Longitudinal study	143; (69, 48,25%)	6	KIDMED	BMI, BIA, skinfolds (triceps, medial calf and front thigh)	-	KIDMED test did not show any association with anthropometric parameters such as BMI, skinfolds, or %FM KIDMED showed a significant correlation with BMI percentiles
Archero F. et al. 2018	2017; Italy	Cross sectional study	669; (324; 48,4%)	6 to 16	KIDMED—Italian version	BMI	-	The KIDMED score did not correlate with BMI, and zscore BMI The risk of OW/OB was not associated with overall adherence to MD. However, associations were observed depending on ethnicity
Bacopoulou F. et al. 2017	2013–2014; Greece	Cross sectional study	1032; (430, 48,70%)	12 to 17	KIDMED	Weight, height, WC, waist-to-height-ratio	WC, SBP and DBP	Post intervention, the percentages decreased to 18.5% for OW and 5.5% for OB WC decreased as the KIDMED score increased (*p* = 0.020)
Calatayud Sáez F. et al. 2011	2008–2011; Spain	Intervention study No control group	98; (55, 56%)	2 to 14	KIDMED	BMI, skinfolds; arm, waist and pelvis circumferences	-	The proportion of FM decreased, and KIDMED score was optimal in 95% of cases after the intervention The observed decrease in BMI is clinically relevant, with a decrease in the proportion of OW and OB. Moreover, the percentage of body FM decreased The KIDMED score increased to ± 3.86 points after intervention
de Santi M. et al. 2020	2017–2018; Italy	Cross sectional study	239; (119, 49,80%)	11 to 13	KIDMED	BMI	-	Adherence to the MD was higher in NW adolescents compared to those with OW and OB (p < 0.001). 53.1% of NW and 85.7% of OB children had low adherence to the MD
Farajian P.et al. 2011	2009; Greece	Cross sectional study	4786; (2359, 49,3%)	10 to 12	KIDMED	BMI, waist and hip circumferences, waist to hip and waist to height ratios, percentages of body fat and body fat mass	-	No differences in KIDMED scores were observed between children with NW and those with OW/OBs (mean difference = 1.08 *p* = 0.28)
Galan-Lopez P. et al. 2019	N/A; Estonia	Cross sectional study	413; (233, 56%)	13 to 16	KIDMED	BMI, body fat percentage, WC and waist perimeter	-	Analysis based on BMI showed a trend to significance, but only in boys (*p* = 0.053) According to levels of adherence to the MD, no statistically different prevalence was observed for non-OW, non-overfat or non-overwaist. However, in boys, participants with low adherence to the MD compared to those with mid-level adherence to the MD were more likely to have overweight
Galan-Lopez P. et al. 2020	N/A; Iceland	Cross sectional study	387; (209, 54%)	13 to 16	KIDMED	BMI, percentage of body fat, WC	-	An inverse association was found between adherence to the MD and BMI (*p* = 0.045) and WC (*p* = 0.029) Differences in KIDMED scores were found between participants with very low BMI and very high BMI (*p* = 0.006)
Galan-Lopez P. et al. 2018	N/A; Iceland	Cross sectional study	387; (209, 54%)	13 to 16	KIDMED	BMI, percentage of body fat, WC	-	When comparing body composition in relation to the degree of adherence to MD, boys showed a higher % of BF among those who had a low adherence (21.84%), compared toto a medium (16.79%) or high adherence (16.21%) (*p* = 0.006)
Galan-Lopez P. et al. 2019	N/A; Spain, Iceland, Estonia	Cross sectional study	1717; (900, 52,41%)	13 to 16	KIDMED	BMI, percentage of body fat, WC	-	Adherence to the MD did not influence OW, over fat and over-WC
Galan-Lopez P. et al. 2019	N/A; Spain	Cross sectional study	917; (458, 49,90%)	13 to 16	KIDMED	BMI, percentage of body fat and WC	-	When comparing body composition parameters (BMI, WC and percentage of BF) according to the degree of adherence to the MD, differences were not observed
Kanellopoulou A. et al. 2020	2014–2016; Greece	Cross sectional study	1142; (502, 44%)	10 to 12	KIDMED	BMI	-	Children with OW/OB had a lower KIDMED scores compared to normal-weight children An inverse association was found between KIDMED scores and weight status
Katsagoni C. N. et al. 2019	2014–2015; Greece	Cross sectional study	174,209; -	6 to 18	MediLIFE-index and KIDMED	BMI, and body mass	-	Students with higher MD scores had lower BMI and WC Those highly adherent to the MD were associated with a lower likelihood of having overweight, obesity, or abdominal obesity compared to those non-adherents
Korkmaz G. O. et al. 2020	2016–2017; Turkey	Cross sectional study	900; (455, 50,60%)	6 to 9	KIDMED	BMI, WC, neck circumference	-	An inverse correlation was found between KIDMED scores and body weight, BMI, WC and neck circumference
Mistretta A. et al. 2016	2012–2014; Italy	Cross sectional study	1643; (885, 53,90%)	11 to 16	KIDMED	BMI, WC, body fat %, fat mass, fat free mass, total body water, FBW	WC, SBP and DBP	KIDMED scores were lower among individuals with OW/OB compared to individuals with normal-weight Good adherence to the MD resulted in a 29% decreased odds of having OW or OB (OR 0.70, 95% CI:0.56–0.87) in both boys and girls An inverse association was found between the KIDMEDscore and BMI (β = -0.042 and β = -0.063 for boys and girls), same with WC (β = -0.012 vs β = -0.011) and FM (β = -0.036 vs β = -0.028)
Munrakami K. et al. 2016	1997; Great Britain	Cross sectional study	1617; (822, 50,80%)	4 to 18	MDS	BMI, BMI z score, WC, and Waist to height ratio (> 11 y)	-	Meals and snacks were inversely associated with certain healthy food groups characteristic of the Mediterranean dietary pattern (vegetables, fruits, cereals) and positively associated with less healthy foods (biscuits/cakes/biscuits, saturated fats, etc.). However, no significant associations were observed between energy density and adiposity measures after adjustment
Munrakami K. 2018	1997; Great Britain	Cross sectional study	1617; (822, 50,80%)	4 to 18	MDS	BMI, BMI z score, WC, and Waist to height ratio (> 11 y)	-	Higher FSA scores (indicating lower nutritional quality) of meals were inversely associated with overall diet quality assessed by the MD score in both children and adolescents FSA scores of meals based on time were inversely associated with z-score BMI in children, whereas those of snacks based on time showed a positive association
Notario-Barandiaran L. et al	2003–2008; Spain	Longitudinal study	1801; (937, 52%)	4 to 8	Rmed	BMI, and WC	-	At the age of 4 years, no association was observed between adherence to the MD and OW, OB, or abdominal OB. However, in longitudinal analyses, high adherence to the MD at the age of 4, was associated with a lower incidence of OW; OB, and abdominal OB at the age of eight
Rodriguez Cabrero M. et al. 2012	N/A; Spain	Cross sectional study	1057; -	14 to 15	KIDMED	BMI	-	There was no relationship between KIDMED score and body composition
Rosa Guillamón A. et al. 2019	N/A; Spain	Retrospective, descriptive, and cross-sectional study	520; (219, 42,20%)	8 to 17	KIDMED	BMI	-	The MD was not significantly associated with weight status Schoolchildren with NW had an adherence to the MD than their peers with OW
Marcos-Serrano M. et al. 2016	N/A; Spain	Cross sectional study	233; (116, 49,80%)	9 to 12	KIDMED	BMI, body mass, fat mass %, fat mass, free fat mass, bone weight, Waist-to-hip ratio, and Lean body mass	Glucose, cholesterol, and triglycerides	No differences were found in anthropometric parameters among students with different KIDMED scores
Tognon G. et al. 2013	2007–2008; Belgium, Cyprus, Estonia, Hungary, Italy, Spain and Sweden	Longitudinal study	14,972; (7621, 50,90%)	2 to 9	fMDS	BMI, WC, triceps, and subscapular skinfold thickness, % fat mass	-	High scores in fMDS were inversely associated with OW, including OB, and percentage of FM, independently of age, sex, socioeconomic status, study centre and PA High fMDS at baseline was protective against increases in BMI, WC, and WtHR
D. Bučan Nenadić et al. 2021	2019; Croatia	Cross sectional study	598; (310, 51.8%)	3 to 7	KIDMED	BMI, BMI z-score, WC, Mid-upper arm circumference (MUAC)	-	Adherence to the MD has shown beneficial effects on body composition and protection against OB in all age groups
M.D.M. Fernández-Álvarez et al. 2021	2018; Spain	Cross sectional study	303; (303, 100%)	13 to 16	KIDMED	BMI and BMI z-score	-	Lower KIDMED scores were associated with excess weight (*p* = 0.032)
R. Fernández-Iglesias et al. 2021	2018; Spain	Cross sectional study	309; (151, 48.9%)	8 to 13	KIDMED	BMI and BMI z-score	-	Optimal adherence to the MD through KIDMED did not show associations with BMI
L.P. Gallardo et al. 2021	2008–2019; Spain	Descriptive transversal	314; (182, 58%)	6 to 9	KIDMED	BMI	-	The KIDMED score did not show associations with body composition variables, neither when stratifying by sex
E.S. George et al. 2021	2007–2009; Spain	Cross sectional study	1972; (990, 50.2%)	9 to 13	KIDMED	BMI	SBP, DBP, plasma glucose, fasting glucose, fasting insuline, serum insuline, TG, HDL, LDL, HOMA-IR	Poor adherence to the MD was associated with central OB (OR: 1.31; 95% CI 1.01–1.73), assessed by measuring WC, complementing the assessment of nutritional status with BMI classification
M.C. Martíncrespo-Blanco et al. 2022 **	2017; Spain	Randomized Trial	133; (67, 50.4%)	3 to 5	KIDMED	BMI	-	In the experimental group, a reduction in BMI was found A statistical trend was found between groups (*p* = 0.076) in the overall KIDMED score. A 10-week intervention was conducted, with 9 participants in the control group receiving 40-min weekly sessions focused on educating them about the human body and health. The experimental group received 45-min weekly sessions, where children were educated about food production, the importance of a varied diet, adequate food consumption according to the healthy food pyramid, and an understanding of the origin and frequency of consumption of various foods such as fruit, vegetables, milk, fish, meat and legumes was fostered
M. Seral-Cortes et al. 2021	N/A; Austria, Belgium, France, Germany, Greece, Hungary, Italy, Spain and Sweden	Cross sectional study	2047; (925, 45.3%)	12.5 to 17.5	MDS	BMI, WC, Subscapular and tricipital skinfold thicknesses, FMI	-	Girls with high adherence to the MD and a lower screen time, were associated with lower BMI, WC and FMI. These findings suggest that the MD may have a protective effect against adiposity, especially in women with lower screen time habits
A. Sümen et al. 2022	2020–2021; Turkey	Cross sectional study	907; (406, 44.8%)	14 to 17	KIDMED	BMI, BMI z-score, WC, hip circumference, neck circumference, waist/hip ratio, Waist/height ratio	-	The KIDMED score was negatively correlated with adolescent’s body weight, neck circumference and BMI (p < 0.005), as well as WC and hip circumference (p < 0.01), and WtHR (p < 0.001)
L.M.N. Sørensen et al. 2021	1999–2008; Norway	Cohorts	52,424; (N/A)	3 to 8	fMDS and DQI	BMI	-	A higher DQI score at the age of 3 was associated with a lower risk of having OW/OB at age the of 8, compared to low DQI scores (OR:0.77; 95%CI: 0.62–0.96), but not with MD adherence
F.Ç. Yılmaz 2021	2019; Turkey	Cross sectional study	367; (175, 47.6%)	10 to 18	KIDMED	BMI, WC	SBP, DBP, HDL, LDL, TG, glucose	Low adherence to the MD group showed high levels of TG and lower levels of DBP compared with medium or high adherence groups. No associations were found with BMI and WC
A.A. Aljahdali et al. 2022	1997–2004; Mexico	Cohort	574; (N/A)	8 to 21	DASH, aMedDiet, C-DII	BMI, WC	SBP, DBP, insuline, serum glucose, TG, HDL, LDL, HOMA-IR	Regarding the aMedDiet score, a positive association was found with HDL-c in boys in the highest quartile of adherence compared to the lowest quartile. No associations were found between adherence to the aMedDiet score and anthropometric and other cardiometabolic risk factors
T.A. Bekelman et al. 2022	2006–2015; U.S	Cohort	581; (288, 49.56%)	10 to 16	HEI-2010, aMED and DASH	BMI, BMI z-score	-	No associations were observed between the aMED score and BMI and z-score BMI in males and females
L. Grams et al. 2022	2015; Spain and Germany	Longitudinal Cohort	334; (168, 50.2%)	10 to 13	KIDMED and IAES	BMI, BMI z-score, Hip circumference	-	In both countries, lower z-score BMI in girls was associated to better diet quality. No associations were observed for the KIDMED score
X. Zheng et al. 2023 *	2005–2018; U.S	Cross-sectional	15,658; (7927, 50.62%)	2 to 19	HEI-2015, AHEI-2010 and MedDiet	BMI	-	A high score in the MD was associated with lower risk of having OW and OB, particularly in male children and adolescents. The HEI-2015 score was related to the risk of having OW in children
B. Kocaadam-Bozkurt et al. 2023 **	2021–2022; Turkey	Cross-sectional	1137; (514, 45.2%)	13 to 15	MSDPS	BMI, BMI z-score, WC, Body fat %	-	MSDPS was associated with BMI and WC (p < 0.005). No associations were observed for z-score BMI and BF %

KIDMED, adherence to Mediterranean diet; MediLIFE-index, Mediterranean Lifestyle Index; *MDS* Mediterranean Diet Score; *Rmed* relative Mediterranean Diet Score; *DQI-A* Diet Quality Index for Adolescents; *HDL-I* Healthy Lifestyle Diet-Index; *MDS* frequency-based Mediterranean Diet Score; *DQI* Diet Quality Index; *DASH* Dietary Approach to Stop Hypertension; aMedDiet, adherence to Mediterranean Diet; *C-DII* Childrens’s Dietary Inflammatory Index; *HEI* Healthy Eating Index; *aMED* alternated Mediterranean Diet; *IAES* Index of a healthy Alimentation diet for the Spanish population; *MSDPS* Mediterranean-Style Dietary Pattern Score; *BMI* Body Mass Index; *BIA* single frequency bioimpedance; *FM* fat mass; *OW/OB* overweight/obesity; *NW* normal weight; *WC* waist circumference; *DBP* diastolic blood pressure; *SBP* systolic blood pressure; *BP* blood pressure; *ED* energy density; *HOMA-IR* Homeostatic Model Assessment Insulin Resistance

**Table 2:** Summary of the findings for different diet quality indices and body composition and metabolic syndrome measures

Author and Publication Year	Recruitment Year; Country	Study Design	Population study (N); Boys (N,%)	Age	Diet Quality Index	Body Composition Measures	Metabolic Syndrome Measures	Summary of main results
Ruopeng An. 2015	2003–2006; U.S	Cross sectional study	2818; (1445, 51,30%)	6 to 17	HEI-2010	BMI	-	Children classified in the healthy diet group (high score of the HEI-2010) presented a lower risk of having OW and OB compared to children with a low HEI-2010 score
Asghari G. et al.2016	1999–2008; Irán	Cohort study	424; (178, 42%)	6 to 18	DASH score	WC	Arterial blood pressure, SBP, DBP, FPG, Serum TG concentrations, HDL-C, WC	A decreasing linear trend for risk of developing MetS was observed with an increase in adherence to the DASH-style diet. This was associated with a decrease in the risk of developing abdominal OB (OR 0.35; 95% IC 0.14–0.89), high FPG (OR 0.40; 95% IC 0.15–0.99) and hypertension (OR 0.30, 95% IC 0.10–0.88)
Berz J. P. B. et al. 2011	1987–1988; U.S	Cross sectional study	2327; only females included	9 to 10	modified DASH score	BMI	-	Adherence to the DASH eating pattern in adolescent girls was associated with smaller increases in BMI over a period of 10 years Total fruit was the strongest predictor of BMI (p < 0.001), followed by low-fat dairy (p < 0.001)
De Miguel-Etayo P. et al. 2019	2007–2009 Spain	Cohort study	117; (51, 43,60%)	13 to 16	DQI-A	BMI, BMI z-score, skinfolds thickness: triceps, biceps, subscapular and suprailiac, FMI	-	Changes in DQI-A were statistically significant associated with changes in z-score BMI and z-score FMI during follow-up
Er V. et al. 2018	2015–2016; United Kingdom	Cross sectional study	150; (72, 48%)	2 to 4	CFT and NAP SACC UK	BMI z-score	-	Adherence to NAP SACC UK (CFT guidelines) was not associated with z-score BMI
Golpour-Hamedani S. et al. 2017	2015–2016; Iran	Cross sectional study	456; (189, 41%)	11 to 18	DASH score	BMI, percentage of body fat, lean body mass, waist and hip circumferences and waist-to-height ratio	-	Higher adherence to the DASH diet was inversely associated with general OB. However, after controlling for confounding factors, this association was attenuated Furthermore, higher adherence to the DASH diet was negatively associated with central OB in children, but this association was not significant
Hajna S. et al. 2012	2007–2008; Canada/U.S	Cross sectional study	1570; (788, 50,20%)	11 to 13	DASH score and Canada’s Food Guide	BMI, waist girth, hip girth, WHR and WHtR	-	In girls, CFG score was associated with lower WHtR, WHR and WG. Further, a higher CFG score was associated with decreased BMI, HG, and risk of OW In boys, no associations were observed between the CFG score and measures of body composition or the risk of OW. In contrast, a higher DASH score was associated with decreased measures of body composition in both sexes The DASH score was negatively associated with BMI, WHtR and WG
Hooshmand F. et al. 2018	2006–2008; Iran	Cohort study	424; (182, 43%)	6 to 18	Modified HEI	WC and BMI	Blood pressure, SBP, DBP, FPG, HDL-C, WC, FBG serum TG	Greater mHEI scores, reflecting improved diet quality, may impede the onset of MetS in children and adolescents
Jiménez-Pavón D. et al. 2013	N/A; Austria, Belgium, France, Germany, Greece, Hungary, Italy, Spain and Sweden	Cross sectional study	637; (290, 45,50%)	12.5 to 17.5	DQI-A and DQI-PA	WC, BMI, skinfolds thickness: triceps, biceps, subscapular, suprailiac, thigh and medial calf, total body fat	Serum concentration of glucose and insulin, HOMA index and QUICKI (quantitative insulin sensitive check index)	In males, there was a direct association between DQI-A and CRF, whereas DQI-PA exhibited a direct association with CRF in both sexes. Furthermore, after adjusting for pubertal status, center, BMI, and CRF, DQI-PA showed an inverse association with HOMA and a direct association with QUICKI in females, but not in males
Lakka T. A. et al. 2020	2007–2009; Finland	Non-randomised controlled trial	504; (261, 51,80%)	6 to 9	FCHEI	BMI, BMI-SDS, Body Fat % and lean body mass	Serum insulin, Plasma glucose and HOMA-IR, Fasting Insulin and Fasting glucose	A combined PA and dietary intervention attenuated the increase in insulin resistance, assessed by fasting serum insulin and HOMA-IR, but had no effect on fasting plasma glucose over 2 years in the general population of children
Linardakis M. et al. 2008	2001–2003; Greece	Cross sectional study	1209; (542, 44,80%)	3 to 17.5	HEI-2010	WC, BMI, WtHR	Blood preasure (SBP and DBP), glucose, Fasting blood glucose, Total cholesterol, LDL and HDL, TC/HDL-C ratio and TG	There was no association between HEI score and clusters of metabolic risk factors Higher diet quality scores were associated with decreased mean BMI, WHtR, SBP, total cholesterol, and other factors associated with MetS
Lioret S. et al. 2014	2007; Australia	Cohort study	216; (95, 44%)	5 to 12	DQI based on Australian Dietary Guidelines	BMI and BMI z-score	-	Improvement in diet quality is associated with a concurrent improvement in z-score BMI among children with OW, but not with children with normal BMI status
Sá Lustosa L. C. R. et al. 2019	N/A; Brazil	Cross sectional study	327; (133, 40,70%)	14 to 19	BHEI-R	WC, BMI and BMI z-score	WC, Blood preasure (SBP and DBP), glucose, blood glucose, HDL-C and TG	Even though the prevalence of MetS was low, notable changes in its components were observed, linked to reduced consumption of important items from the BHEI-R
McGee M. et al. 2020	2016–2018; Canada	Prospective cohort study	158; (84, 46,80%)	2 to 5	HEI-2010	WC, BMI, BMI z score, fat mass, fat-free mass, FMI, fat-free mass index, subscapular and triceps skinfold thickness	-	HEI 2010 scores were inversely associated with BMI, WC, triceps and subscapular z-scores but not with FMI or FFMI
Mohseni-Takalloo S. et al. 2016	2008–2011; Iran	Cross sectional study	722; (330, 45,70%)	10 to 19	DGAI , HEI 2005 and HEI 2010	WC, BMI	Glucose and lipid concentration, fasting blood glucose, triglyceride, HDL-c, blood preasure (SBP and DBP)	Mean values of BMI and WC showed a significant decreasing trend according to quartiles of HEI-2010 score There was an association between DGAI and HEI-2005 scores and HDL-C concentration, an inverse association between HEI-2005 score and fasting blood glucose and triglyceride concentrations Individuals at the highest quartile category of HEI-2010 score reduced the risk of having central OB by 37% (*p* = 0.04) and general OB by 38% (*p* = 0.03) No association was observed between different OB classifications and other diet quality scores
Pan Y. et al. 2008	1999–2002; U.S	Cross sectional study	4450; (2260, 50,80%)	12 to 19	HEI	WC and BMI	WC, Plasma glucose and serum, plasma triglycerides, HDL and blood pressure (SBP and DBP)	Increments of overall HEI score and fruit score quartiles decreased the prevalence of MetS
Perry C. P. et al. 2015	2007–2008; Ireland	Cohort study	8568; (4150, 51.3%)	9	DQS	BMI	-	There was a significant mean difference in the DQS for children having OB compared to children with NW (p < 0.001), and children with OB vs. children with OW (p < 0.001)
Setayeshgar S. et al. 2016	2005–2008; Canada	Cohort study	546; (302, 55%)	8 to 10	DQII	Fat mass, % body fat, % central body fat, FMI, central fat mass index, BMI BMI z-score	-	Each 10-unit improvement in overall DQI score was associated with lower gain in CFMI, and %BF Each unit improvement in dietary adequacy score was associated with a lower gain in FMI, CFMI, %BF, and %CBF
Thomson J. L. et al. 2019	2009–2014; U.S	Cross sectional study	8894; (4538, 50,80%)	2 to 18	HEI-2015	BMI	-	Overall diet quality from HEI-2015 and most of its components did not differ among children with different BMI categories
Torres R. et al. 2014	2010–2011; Puerto Rico	Cross sectional study	796; (362, 45,50%)	12	HEI-2005	BMI	-	This study suggests that the HEI-2005 might not provide a comprehensive understanding of OB, because it only assesses adherence to dietary guidelines without considering other potential factors. Some components of diet quality were associated with social determinants of weight status
Torres R. et al. 2014	2012–2013; Puerto Rico	Cross sectional study	114; (49, 43%)	12	HEI-2010	BMI	-	Diet quality was not associated with weight status Overall HEI-2010 scores were similar in children with NW and OW/OB
Wong J. E. et al. 2014	2011; New Zealand	Cross sectional study	681; (384, 56%)	14 to 18	NZDQI-A	WC, BMI, BMI zscore, fat mass, fat-free mass, WtHR, Fat-to-lean mass ratio, FMI, fat-free mass index and body fat %	-	There were no significant differences in a NZDQI-A scores between males and females or by weight Higher NZDQI-A scores were significantly associated with lower BF%, fat-to-lean mass ratio and lower FMI, after multivariate adjustment No association was found between NZDQI-A and BMI, WC or WHtR ratio
E. Asgari et al. 2022	2017–2018; Iran	Cross sectional study	788; (0, 0%)	6	MIND	BMI, BMI z-score	-	Higher adherence to the MIND diet was associated with a lower odds of having OW and OB in 6 years old Iranian girls
M. Askari et al. 2021	2017–2018; Iran	Cross sectional study	788; N/A	6	HEI-2015	BMI, Mid-arm	-	There was an association between HEI-2015 scores and OW status in 6-year-old children. However, no associations were reported with other anthropometric parameters
P.C. Vinke et al. 2020	2006–2007; Netherlands	Cohorts	1001; (508, 50.7%)	3 to 10	LLDS	BMI and BMI z-score	-	Diet quality measured with LLDS at the age of 3, was associated with changes in z-score BMI and OW incidence between the ages of 3 to 10 years
L.P. Bricarello et al. 2021	2013–2014; Brazil	Cross Sectional Study	71,553; (31,863, 44.53%)	12 to 17	DASH	BMI and BMI z-score	-	No association was found between the DASH diet score and OW/OB in Brazilian adolescents
K. Ducharme-Smith et al. 2021	2015–2020; U.S	Cohorts	90; (51, 56.7%)	4 to 21	AHEI-2010 and C-DII	BMI, BMI z-score, WC	SBP, DBP, Blood pressure index, Plasma glucose, TG, serum HDL, total cholesterol, left ventricular mass index, HbA1c	Participants with higher AHEI-2010 scores and more anti-inflammatory diets trended toward lower SBP, DBP, LVMI, HbA1c and non-HDL cholesterol Participants with anti-inflammatory diets also trended toward lower z-score BMI and z-score WC
K. Ducharme-Smith et al. 2021	2005–2016; U.S	Cross Sectional Study	2459; (1259, 51.2%)	12 to 21	AHEI-2010 and DASH	BMI, BMI z-score, MUAC	SBP, DBP, BP index, Fasting plasma glucose, HDL, TG	No associations were found between the DASH pattern and OW/OB, impaired glucose, low HDL-c or hypertriglyceridemia. Similarly, no significant associations were found between AHEI-2010 scores and individual cardiometabolic components Both scores were negatively associated with the odds of having MetS
P. Latorre-Román et al. 2022	N/A; Spain, Chile and Colombia	Cross Sectional Study	982; (424, 43.2%)	4 to 6	Krece Plus Test	BMI, BMI z-score, WC, Weight status z-score, WtHR	-	Lifestyle factors were associated with AO among Spanish-speaking preschool children, with physical fitness especially being a relevant factor regardless of the country of origin
M. Liu et al. 2021	1997–2009; China	Cohorts	2903; (1533, 52.8%)	7 to 17	mCCDI	WC	SBP, DBP, BP, glucose, HDL and TG	Higher diet quality measured by the mCCDI was independently associated with a lower MetScore (B:-0.11; 95% CI: -0.18- -0.04), and higher lagged mCCDI over time was associate with lower z-score WC (B:-0.05; 95% CI: -0.08- -0.01)
J.L. Pereira et al. 2021	2015; Brazil and U.S	Cross Sectional Study	976; (492, 50.4%)	12 to 16	AHEI-2010 and BHEI-R	BMI, WC	SBP, DBP, Total cholesterol, HDL, LDL, TG, insuline resistence, HOMA-IR, high fasting plasma glucose, HbA1c	BHEI-R was inversely associated with OW/OB (OR:0.87; 95% CI: 0.80–0.95) and cardiovascular risk factors (OR:0.89; 95% CII: 0.80–0.98) A healthier diet quality was associated with lower odds of having OW/OB in Brazilian and USA-Hispanic/latino adolescents. Additionally, it was associated with lower cardiovascular risk in Brazilian adolescents
K. Sahel et al. 2022	2016–2018; Morocco	Cross Sectional Study	463; (194, 58.1%)	9 to 17	DDS and DVS	BMI, BMI z-score, WC and WHTR	-	There was no correlation between the DDS and BMI as well as WHTR, whereas a significant inverse correlation was identified between the DVS and BMI
S. S. Summer et al. 2021	2011–2016; U.S	Cross Sectional Study	1214; (617, 50.8%)	12 to 19	HEI-2015	BMI z-score, Sagital abdominal diameter	SBP, HDL, TG, fasting glucose	A higher HEI-2015 score showed a negative association with z-score MetS, indicating that superior overall diet quality is associated to a decreased risk of MetS
K. Hu et al. 2023 *	2016–2018; U.S	Cross Sectional Study	192; (92, 47.9%)	10 to 16	HEI-2015	BMI z-score, WC, body fat %, lean mass, VAT mass	SBP, DBP, HDL-c, insuline, glucose, HOMA-IR	HEI-2015 score was inversely associated with z-score WC, z-score BMI, FM, lean mass, VAT mass, and z-score HOMA-IR
F. Ali Said et al. 2023	2021–2022; Tanzania	Cross Sectional Study	2556; (1234, 46.7%)	5 to 19	PDQS	BMI, BMI z-score	-	There was a significant association between diet quality assessed by the PDQS and BMI of children and adolescents

*HEI* Healthy Eating Index; *DASH* Dietary Approach to Stop Hypertension; *DQI* Diet Quality Index; *DQI-A* Diet Quality Index for Adolescents; CFT and NAP SACC UK Children's Food Trust and Nutrition Best Practice Standards; *DQI-PA* Diet Quality Index and Physical Activity; *FCHEI* Finland Children Healthy Eating Index; *BHEI-R* Brazilian Healthy Eating Index Revised; *DGAI* Dietary Guidelines for Americans Adherence Index; *DQS* Dietary quality score; *DQII* Diet Quality Index – International; *NZDQI-A* New Zeland Diet Quality Index for Adolescents; *MIND* Mediterranean-DASH Intervention for Neurodegenerative Delay; *LLDS* Lifelines Diet Score; *aHEI* alternative Healthy Eating Index; *C-DII* Children’s Dietary Inflammatory Index; *mCCDI* modified version of the Chinese Children Dietary Index; *DDS* Dietary Diversity Score; *DVS* Food Variety Score; *PDQS* Prime Dietary Quality Score; *MetS* metabolic syndrome; *BMI* Body Mass Index; *PA* Physical Activity; *OB* obesity; *OW* overweight; *NW* normal weight; *SBP* systolic blood pressure; *DBP* diastolic blood pressure; *FPG* fasting plasma glucose; *FBG* fasting blood glucose; *TG* triglycerides; *HDL-C* high density lipoprotein-cholesterol; *WC* waist circumference; *FMI* Fat Mass Index; *WG* waist girth; *WHR* waist height ratio; *WHtR* waist to height ratio; *CRF* cardiorespiratory fitness; *CFMI* central fat mass index; *HOMA-IR* Homeostatic Model Assessment Insulin Resistance; *HbA1c* Haemoglobin A1

Overall, thirty eight studies (52%) were conducted in European countries [15, 16, 28–63], ten studies (14%) were conducted in Asian countries [64–73], two studies (3%) were conducted in Australia [74] and New Zealand [75], and one study was conducted in Morocco [76]. The remaining studies were carried out in the Americas, with 29% conducted in U.S and Canada [18, 77–89], and Latin America (including Caribbean) [17, 90–95]. Additionally, one study was conducted in Africa [96]

The studies were evaluated as cross sectional (*n* = 53, 72%), cohort (*n* = 15, 22%), and clinical trials (*n* = 4, 6%).

### Tools Used to Assess Dietary Quality Scores in the Studies

#### Mediterranean Diet Scores

A total of 37 studies examined the Mediterranean dietary pattern. Among these, 25 used the KIDMED score, with one study considering the Italian version [38]. Of these studies, 11 were conducted in Spain [40–42, 45–47, 50–52, 54, 57], 3 in Greece [29, 49, 58], 3 in Turkey [67, 69, 70], 2 in Italy [33, 60], and 5 in other European countries [39, 48, 59, 62, 63]. Additionally, other DQSs related to the Mediterranean dietary pattern were identified. Three articles used the Mediterranean Diet Score (MDS) [34, 35, 55], while the Relative Mediterranean Diet Score (rMDS) and the Frequency-Based Mediterranean Diet Score (fMDS) were each used in one study [37, 43]. One study used both the fMDS and a Diet Quality Index (DQI) [97], while another study used three scores: aMedDiet, Dietary Approaches to Stop Hypertension (DASH) and Childrens’s Dietary Inflammatory Index (C-DII) [92]. One study used both the KIDMED score and the MedLIFE-index [30]. Further, one study used the KIDMED score alongside with two other dietary scores (DQI-A and Healthy Lifestyle Diet-Index (HDL-I)) [44]. Another study used the KIDMED and an Index of a healthy Alimentation diet for the Spanish population (IAES) [53]. Moreover, one study used a combination of the Healthy Eating Index-2015 (HEI), Alternative HEI-2010 (AHEI) and MedDiet [81], while another study used the Mediterranean-Style Dietary Pattern Score (MSDPS) [73].

#### Other Diet Quality Scores

Different versions of the HEI were used in 17 studies: one study used the HEI [18], one study used the HEI-2005 [90], 4 studies used the HEI-2010 [32, 77, 85, 91], and 4 studies used the HEI-2015 [72, 78, 80, 83]. Moreover, adaptations of the HEI were also employed: one study utilized the Modified HEI [66], another used the Finland Children Healthy Eating Index (FCHEI) [31], and one study employed the Brazilian Healthy Eating Index Revised (BHEI-R) [17]. Additionally, one study used both HEI-2005, HEI-2010, along with the Dietary Guidelines for American Adherence Index (DGAI) [68]. Another study used the AHEI-2010 and the C-DII [88], while another study used the AHEI-2010 and DASH [89]. One study combined the AHEI-2010 and the BHEI-R [5].

DQI was employed in 7 studies. Among these, 4 studies used the DQI for Adolescents (DQI-A). Specifically, one used the DQI-A [16], another study also used the DQI including Physical Activity (DQI-PA) [28], and a third study used the New Zealand DQI-A (NZDQI-A) [75]. Additionally, one study used three types of DQSs: KIDMED, DQI-A and HDL-I [44]. The other DQI studies used two different versions of the score: the DQI based on Australian Dietary Guidelines [74] and the DQI-International (DQII) [86]. Finally, one study used the Prime Dietary Quality Score (PDQS) [96].

Regarding the DASH score, 5 studies used this score: 3 studies employed the DASH score [64, 65, 93], one study used the Modified DASH score [82] and another study used both the DASH score and the Canada’s Food Guidelines (CFG) [84].

Seven additional studies using different DQSs were identified. One study used the validated Dietary Quality Score (DQS) [36] while another considered the Children’s Food Trust (CFT) and the Nutrition and Physical Activity Self-Assessment for Child Care UK Nutrition Best Practice Standards (NAP SACC UK) [61]. Furthermore, the Mediterranean-DASH Intervention for Neurodegenerative Delay (MIND) was employed in another study [71]. The Lifelines Diet Score (LLDS) [56], the Krece Plus Test [94], and the modified version of the Chinese Children Dietary Index (mCCDI) [79] were each used in separate studies. Finally, one study combined the use of Dietary Diversity Score (DDS) and food variety score (DVS) [76].

### Obesity Assessment

Most of the studies found were assessing associations with obesity outcomes (*n* = 52; 73%). All the studies used the measures of weight, height and BMI as obesity related measures. Furthermore, 22 out of 52 also included WC as an obesity-related outcome. Some studies combined BMI and WC with other body composition measurements, such as skinfolds [16, 37, 40, 41, 55, 85] or body fat percentage [30, 39, 42, 58, 59, 62, 63, 65, 73, 75, 80, 84, 86].

### Metabolic Syndrome Assessment

A total of 21 studies (29%) measured MetS. All the studies used outcomes such as abdominal obesity, insulin resistance, hypertension, and dyslipidemia, except for 3 studies [28, 31, 47]. Additionally, total cholesterol and triglycerides were measured in some studies [17, 18, 32, 44, 47, 52, 64, 66, 68, 70, 78, 79, 88, 89, 92, 95], and six studies considered the HOMA index [28, 31, 52, 80, 92, 95].

### Meta-Analysis Results

**Fig. 2:**
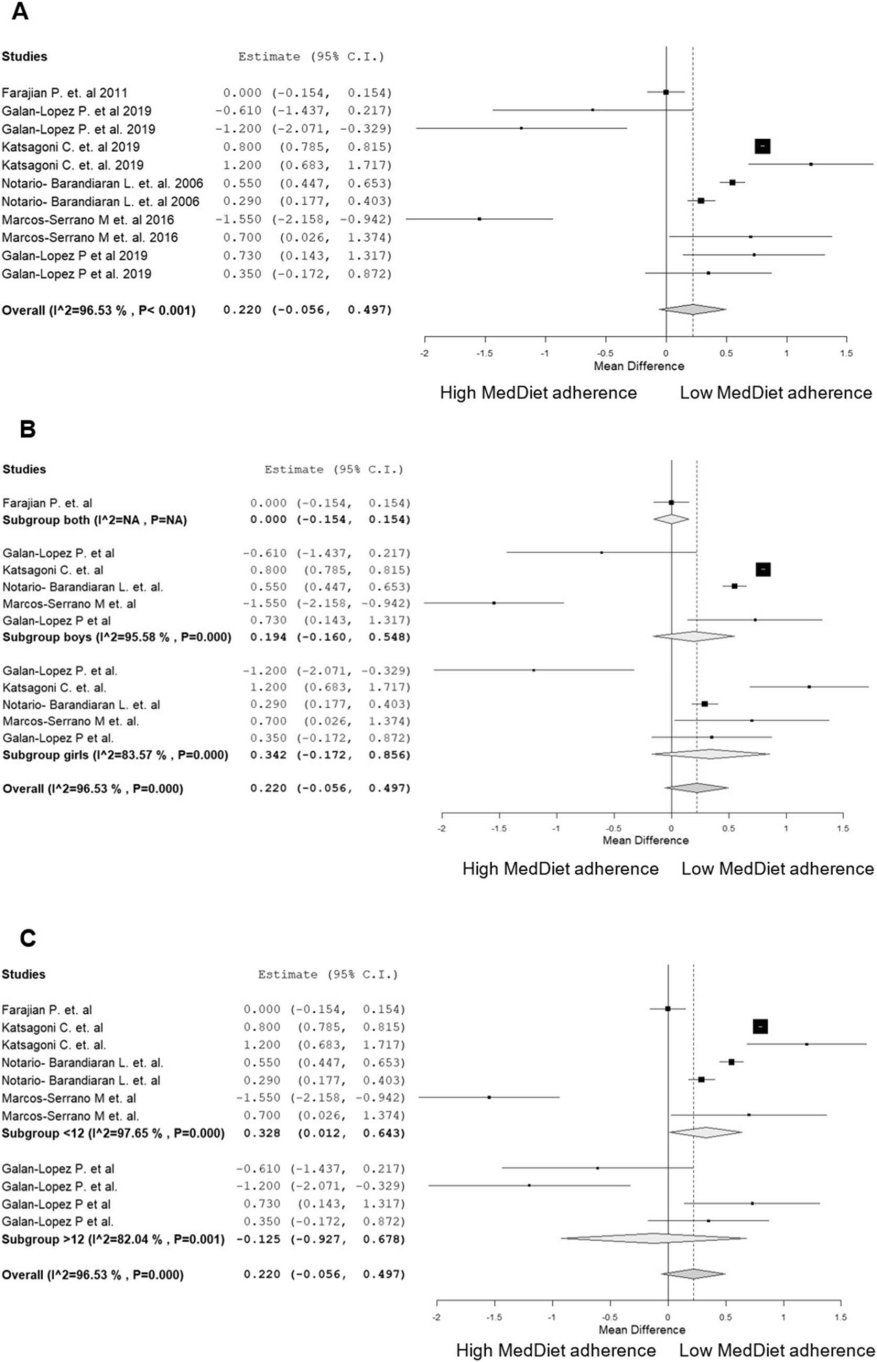
Random-effects meta-analysis of the effects of low adherence versus high adherence to the MD scores on BMI. **A** BMI differences between low and high adherence to the MD scores. **B** Subgroup analyses by sex. **C** Subgroup analyses by age. Abbreviations: MD, Mediterranean diet; BMI, Body Mass Index

**Fig. 3:**
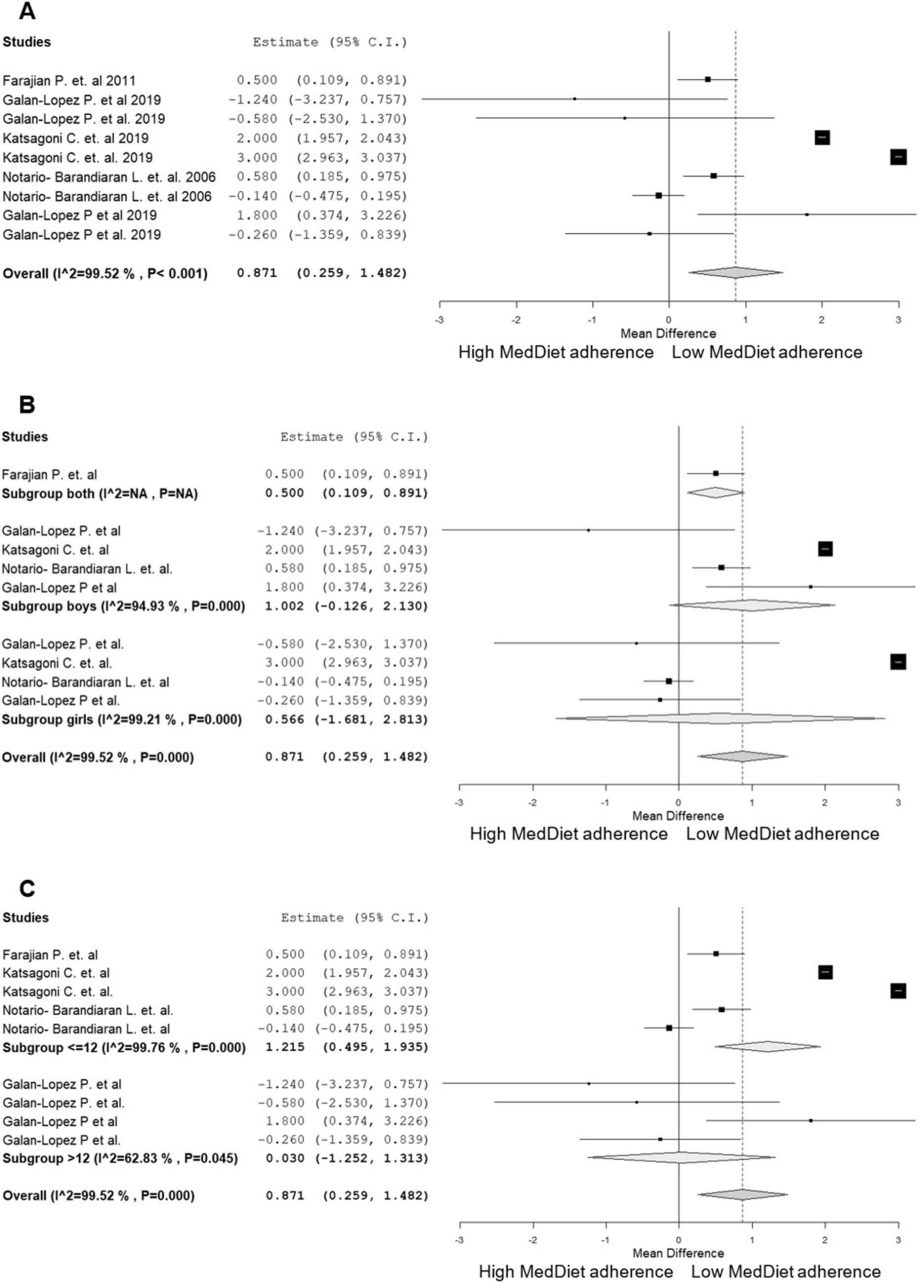
Random-effects meta-analysis of the effects of low adherence versus high adherence to the MD scores on WC. **A** WC differences between low and high adherence to the MD scores. **B** Subgroup analyses by sex. **C** Subgroup analyses by age. Abbreviations: MD, Mediterranean diet; WC, Waist Circumference

The meta-analysis was comprised of data from six original articles that conducted analyses with 5 separate cohort studies: GRECO (Greece); EYZHN (Greece); INMA (Spain); AdolesHealth (Estonia, Iceland and Spain); and an additional cross-sectional sample of school children from Spain. However, it is important to note that the AdolesHealth study involved participants from Estonia in one study and participants from Estonia, Iceland and Spain in another study. These studies provided comparable information to perform a meta-analysis [30, 43, 47, 58, 59, 63]. Figures 2 and 3 shows the individual study results and plot the global effect of adherence to the Mediterranean Diet and BMI and WC.

As shown in Fig. 2A, there are no statistically significant differences in the BMI of children with a high adherence to the MD score compared to those with a low adherence score (MD = 0.22 kg/m^2^, 95% CI: -0.06, 0.50). However, due to high variability among the included studies (96% heterogeneity), a trend is observed suggesting that children with a high MD score adherence tend to have a lower BMI than those with a low MD score adherence. In subgroup analysis by sex (Fig. 2B), no statistically significant differences are shown in the BMI of children with a high MD score adherence compared to those with a low MD score adherence (MD = 0.19 kg/m^2^, 95% CI: -0.16, 0.55). Regarding age, (Fig. 2C), statistically significant differences in BMI are observed among children younger than 12 years old, favouring those with a high MD score adherence over those with a low MD score adherence (MD = 0.33 kg/m^2^, 95% CI: 0.01, 0.64). However, in children older than 12 years old, no statistically significant differences are observed in BMI between those with a high MD score adherence and those with a low MD score adherence (MD = -0.13 kg/m^2^, 95% CI: -0.09, 0.68). Nevertheless, considering the high proportion of heterogeneity among the studies included in the meta-analysis (97% heterogeneity), these differences may not be clinically relevant.

For WC, as shown in Fig. 3A, there are statistically significant differences favouring children with a high MD score adherence compared to those with a low adherence to the MD score (MD = 0.87 cm, 95% CI: 0.26, 1.48). Subgroup analysis by sex (Fig. 3B), does not reveal statistically significant differences in WC between children with high MD score adherence and those with a low adherence score (MD = 1.00 cm, 95% CI: -0.13, 2.13). However, subgroup analysis by age (Fig. 3C), shows statistically significant differences in WC among children younger than 12 years old, favouring those with high MD score adherence over those with low adherence scores (MD = 1.22 cm, 95% CI: 0.50, 1.94). No statistically significant differences are found among children older than 12 years old between those with high adherence scores and those with low adherence scores (MD = 0.03 cm, 95% CI: -1.25, 1.31).

A meta-regression analysis was performed to assess the potential effect of sex and age on the association between adherence to the MD score and BMI, z-score BMI, and WC (Table 3). In children older than 12 years old, the association between adherence to the MD score and BMI, as well as WC, is stronger (β = 21.77, 95% CI: 18.79, 20.67 and β = 72.02, 95% CI: 70.50, 73.54, respectively), compared to younger children. In addition, boys show stronger associations between MD score adherence and BMI, z-score BMI and WC (β = 19.82, 95% CI: 17.62, 22.03, β = 0.64, 95% CI: 0.32, 0.95 and β = 67.03, 95% CI: 57.29, 76.77, respectively) compared to girls.

**Table 3:** Meta-regression analysis of the predictors of having a high MD adherence score

Predictor	β coefficient	95% CI
BMI	19.732	18.79–20.764
Grouped by Sex		
Boys Girls	19.823 19.648	17.621–22.025 17.402–21.894
Grouped by Age		
< 12 y > 12 y	16.924 21.774	16.662–17.186 18.790–20.674
BMI z-score	0.369	0.22–0.518
Grouped by Sex		
Boys Girls	0.635 0.575	0.319–0.951 0.223–0.928
WC	65.748	62.011–69.485
Grouped by Sex		
Boys Girls	67.026 64.499	57.285–76.767 55.824–73.174
Grouped by Age		
< 12 y > 12 y	56.212 72.020	55.232–57.192 70.499–73.541

*BMI* Body Mass Index; *WC* Waist Circumference

## Discussion

The current systematic review and meta-analysis offers a comprehensive summary of the relationship between diet quality scores (DQSs), which have been developed to assess the overall diet quality, and their relationship with obesity and MetS during childhood and adolescence. The MD scores were the most widely used scores to assess the overall diet quality and its association with obesity in children. The main findings of the meta-analysis indicate that high adherence to the MD was associated with low obesity measures but only in adolescents. While we did not perform a meta-analysis for MetS outcome, the published literature suggests that high adherence to a DQS has beneficial effects on MetS and its components in children and adolescents.

Our systematic review identified 25 studies assessing the association between MD adherence using the KIDMED index and obesity measures such as BMI, z-score BMI, and WC. Among these, six articles did not found a significant association between MD adherence and various obesity measures, such as height, weight, BMI and WC. This lack of association could be due to the inclusion of young children. It is more common for associations between MD scores and obesity outcomes tend to emerge more frequently at older ages, as proper adherence to the MD, and consequently its effectiveness, requires time to manifest. Hence, monitoring changes in children´s adiposity is essential for understanding the development of metabolic diseases [98]. These results are consistent with findings in adults, which suggest that greater adherence to the MD may have a protective effect against cardiovascular disease and the development of obesity [99].

Regarding the 5 studies using adapted versions of the MDS, the study using the fMDS found an inverse association between the total score and measures of overweight, obesity and fat mass percentage. An increased fMDS score was found to be a protective factor against high BMI, WC and waist-to-hip-ratio. The study using the rMDS showed an association between high adherence to the MD score at the age of four years old and a low incidence of overweight, obesity or abdominal obesity [43]. This significant association may be attributed to the modified nature of the score used, adapted to the dietary habits of children and adolescents, and calculated based on sex and age [100]. Moreover, the food frequency questionnaires employed in the study covered a longer period of time than other dietary assessment methods, as they involved 24-h dietary recalls. These findings align with those observed in adults, further strengthening the evidence for the MD in preventing overweight and obesity in early childhood.

In this systematic review, only 4 studies investigated the association between MD scores and MetS [33, 44, 47, 49]. This finding may be due to the broader use of the DASH dietary recommendations are more commonly used in preventing the onset of MetS compared to MD recommendations [101]. Concerning the DASH diet, three studies included in this systematic review found an association between the DASH diet and obesity; participants adhering to the diet showed lower obesity measures [64, 65, 84]. One of the studies using the DASH score [64] suggested that the risk of MetS decreases with increasing adherence to the DASH diet.

In relation to the HEI, we found several articles that investigated its association with obesity and MetS. Of the six articles that focused on both diseases, four of them reported that as the HEI score, and its versions, increase, the risk of developing MetS decreases. This finding may be due to the HEI being based on the recommendations of the Dietary Guidelines for Americans, which provide guidance on preventing diet-related chronic diseases, such as cardiovascular disease [102]. In the case of articles associating different versions of HEI with obesity, there are less favourable results for weight status, although three of them found inverse significant associations between HEI scores and the prevalence of overweight or obesity.

Of the six included versions of the DQI included in this systematic review, only one article, which utilized the DQI-A and DQI-PA [28] investigated the association with MetS. The remaining five studies focused on obesity; four of them reported that higher adherence to the DQI was associated with reduced obesity outcomes, such as z-score BMI or body fat percentage. Moreover, The DQI-I, has been adapted for use in a range of countries with different dietary patterns [103]. However, the mentioned study that used an adapted version of the DQI-I according to the Australian dietary guidelines did not find the expected associations between the adapted score and obesity measures. As suggested in a recent systematic review, the construction of population-based DQSs in Australia and New Zealand should be adjusted to align with the original scoring systems [104]. This is particularly important given that nutritional provision in Australian child care settings often fails to comply with national nutritional guidelines for children in early childhood settings [105].

The meta-analysis conducted in this investigation identified significant associations between higher adherence to MD scores and lower obesity measures. Specifically, for both BMI and WC, statistically significant associations favouring higher MD scores were observed among children younger than 12 years old. This finding may suggest that the effects of consuming foods traditionally included in the MD may require more time to manifest during childhood [106]. In addition, it is important to consider the interplay between MD and other lifestyle behaviours (i.e., eating behaviors, physical activity) in terms of obesity development [55].

This systematic review and meta-analysis present some limitations. First, there is a variety of DQSs used to assess diet in the same population, which may introduce heterogeneity into the analyses. Second, some of the studies included in this systematic review did not investigate the association between all obesity measurements, thus limiting the results presented in the meta-analysis and making it challenging to draw conclusions regarding body composition and its association with MD, because the most frequently used obesity measures were BMI and WC. Third, this review did not include grey literature such as conference proceedings, doctoral theses, and technical reports, which could also prove valuable insights. Additionally, although many studies showed high quality (44%), a substantial proportion of them had medium to low quality (48%). Nevertheless, the studies included in the meta-analysis were of high and moderate quality. Most of the included studies were cross-sectional, making it difficult to establish causality. However, our systematic review included a few prospective studies and clinical trials which may reduce the risk of reverse causation. Finally, it is worth noting that the majority of the reviewed studies were conducted among European samples, therefore, the generalizability of these findings to other populations should be evaluated.

A major strength of this evidence synthesis is the comprehensive assessment of studies examining the association between DQSs and obesity and MetS, providing an updated overview of the literature. This review followed strict procedures to ensure the validity of the results. It was registered in the PROSPERO database, followed the PRISMA protocol, involved two reviewers in the development process, conducted quality evaluations of the included studies, and performed a meta-analysis.

Based on the findings of this investigation, an important consideration for future research in this area is the standardization of DQSs. Unifying DQSs would allow for a more uniform assessment of the relationship between diet quality and various parameters used to assess obesity and MetS. This could improve comparability between studies and facilitate a better understanding of the impact of diet quality on health outcomes in children and adolescents.

## Conclusions

This systematic review and meta-analysis suggest that high adherence to diet quality scores has a positive effect on obesity measures and MetS in children and adolescents, although a few studies found no associations between diet quality and the studied outcomes. Specifically, adherence to MD aligns with existing evidence in adults, reinforcing its protective effect against obesity and cardiovascular disease.

The use of DQSs in pediatric populations is becoming more widespread in epidemiology. While DQSs play a role in observational studies, they are also emerging as valuable tools in clinical and interventional studies. However, more research is needed to draw stronger conclusions about the risk of developing health problems in the pediatric population in relation to the different DQSs. Efforts to standardize these scores are necessary to identify those most useful for effectively screening obesity risk.

Future research should focus on prospective study designs and randomized controlled trials to establish causality and provide stronger evidence. Moreover, expanding research beyond European populations would enhance the generalizability of findings to broader demographic groups.

## Key References

54. •• Martíncrespo-Blanco MC, Varillas-Delgado D, Blanco-Abril S, Cid-Exposito MG, Robledo-Martín J. Effectiveness of an Intervention Programme on Adherence to the Mediterranean Diet in a Preschool Child: A Randomised Controlled Trial. Nutrients. 2022;14(8).
  It provides relevant results from an intervention study aiming to increase adherence to the Mediterranean diet in preschool children.

73. • Kocaadam-Bozkurt B, Karaçil Ermumcu MŞ, Erdoğan Gövez N, Bozkurt O, Akpinar Ş, Mengi Çelik Ö, et al. Association between adherence to the Mediterranean diet with anthropometric measurements and nutritional status in adolescents. Nutr Hosp. 2023;40(2):368–76.
  It provides updated contribution to understand the association between adherence to the Mediterranean diet and body composition parameters in adolescents.

80. •• Hu K, Button AM, Tate CM, Kracht CL, Champagne CM, Staiano AE. Adolescent Diet Quality, Cardiometabolic Risk, and Adiposity: A Prospective Cohort. J Nutr Educ Behav. 2023;55(12):851–60.
  It provides longitudinal information on the influence of dietary quality on the development of the metabolic syndrome in adolescents.

81. Zheng X, Wang H, Wu H. Association between diet quality scores and risk of overweight and obesity in children and adolescents. BMC Pediatr. 2023;23(1):1–8.
  It is an important reference that showed that a high Mediterranean diet score is associated to lower risk of overweight/obesity, specially in males.

## Supplementary Information

Below is the link to the electronic supplementary material.

Supplementary file1 (DOCX 35 KB)

Supplementary file2 (DOCX 18 KB)

## Data Availability

No datasets were generated or analysed during the current study.

## References

[1] Bhurosy T, Jeewon R. Overweight and obesity epidemic in developing countries: a problem with diet, physical activity, or socioeconomic status? Sci World J. 2014;2014:1. 10.1155/2014/964236 PMC4212551 25379554

[2] Caballero B. The global epidemic of obesity: an overview. Epidemiol Rev. 2007;29(1):1–5. 17569676 10.1093/epirev/mxm012

[3] Reid M, Worsley A, Mavondo F. The obesogenic household: Factors influencing dietary gatekeeper satisfaction with family diet. Psychol Mark. 2015;32(5):544–57.

[4] Barbalho SM, Oshiiwa M, Sato Fontana LC, Ribeiro Finalli EF, Paiva Filho ME, Machado Spada AP. Metabolic syndrome and atherogenic indices in school children: A worrying panorama in Brazil. Diabetes Metab Syndr. 2017;1(11 Suppl 1):S397-401. 10.1016/j.dsx.2017.03.024 28284912

[5] Pereira AR, Oliveira A. Dietary interventions to prevent childhood obesity: A literature review. Nutrients. 2021;13(10):1–17. 10.3390/nu13103447 PMC8537925 34684448

[6] Kaur J. A comprehensive review on metabolic syndrome. Cardiol Res Pract. 2014;2014:1. 10.1155/2014/943162 PMC3966331 24711954

[7] Noubiap JJ, Nansseu JR, Lontchi-Yimagou E, Nkeck JR, Nyaga UF, Ngouo AT, et al. Global, regional, and country estimates of metabolic syndrome burden in children and adolescents in 2020: a systematic review and modelling analysis. Lancet Child Adolesc Heal. 2022;6(3):158–70. 10.1016/S2352-4642(21)00374-6 35051409

[8] Alkerwi A. Diet quality concept. Nutrition. 2014;30(6):613–8. 24800663 10.1016/j.nut.2013.10.001

[9] Dalwood P, Marshall S, Burrows TL, McIntosh A, Collins CE. Diet quality indices and their associations with health-related outcomes in children and adolescents: an updated systematic review. Nutr J. 2020;19(1). 10.1186/s12937-020-00632-x. 10.1186/s12937-020-00632-x PMC7585689 33099309

[10] Colby S, Zhou W, Allison C, Mathews AE, Olfert MD, Morrell JS, et al. Development and validation of the short healthy eating index survey with a college population to assess dietary quality and intake. Nutrients. 2020;12(9):1–24. 10.3390/nu12092611 PMC7551037 32867172

[11] Gil Á, de Victoria EM, Olza J. Indicators for the evaluation of diet quality. Nutr Hosp. 2015;31(Suppl 3):128–44. 25719781 10.3305/nh.2015.31.sup3.8761

[12] Wang J, Masters WA, Bai Y, Mozaffarian D, Naumova EN, Singh GM. The International Diet-Health Index: a novel tool to evaluate diet quality for cardiometabolic health across countries. BMJ Glob Heal. 2020;5(7):e002120. 10.1136/bmjgh-2019-002120 PMC7375435 32694217

[13] Jennings A, Welch A, Van Sluijs EMF, Griffin SJ, Cassidy A. Diet quality is independently associated with weight status in children aged 9–10 years. J Nutr. 2011;141(3):453–9. 21270356 10.3945/jn.110.131441

[14] Okubo H, Crozier SR, Harvey NC, Godfrey KM, Inskip HM, Cooper C, et al. Diet quality across early childhood and adiposity at 6 years: the Southampton Women’s Survey. Int J Obes (Lond). 2015;39(10):1456–62. 26121960 10.1038/ijo.2015.97 PMC4597330

[15] Sørensen LMN, Aamodt G, Brantsæter AL, Meltzer HM, Papadopoulou E. Diet quality of Norwegian children at 3 and 7 years: changes, predictors and longitudinal association with weight. Int J Obes (Lond). 2022;46(1):10–20. 34462565 10.1038/s41366-021-00951-x

[16] De Miguel-Etayo P, Moreno LA, Santabárbara J, Martín-Matillas M, Azcona-San Julian MC, Marti del Moral A, et al. Diet quality index as a predictor of treatment efficacy in overweight and obese adolescents: The EVASYON study. Clin Nutr. 2019;38(2):782–90. 29730135 10.1016/j.clnu.2018.02.032

[17] de SáLustosa LCR, Nascimento LM, de Carvalho Lavôr LC, Gomes KRO, Mascarenhas MDM, Frota KMG. Metabolic syndrome in adolescents and its association with diet quality. Rev Nutr. 2019;32:1–16.

[18] Pan Y, Pratt CA. Metabolic syndrome and its association with diet and physical activity in US adolescents. J Am Diet Assoc. 2008;108(2):276–86. 18237576 10.1016/j.jada.2007.10.049

[19] Garcidueñas-Fimbres TE, Paz-Graniel I, Nishi SK, Salas-Salvadó J, Babio N. Eating speed, eating frequency, and their relationships with diet quality, adiposity, and metabolic syndrome, or its components. Nutrients. 2021;13(5):1687. 34063439 10.3390/nu13051687 PMC8156274

[20] Caferoglu Z, Erdal B, Hatipoglu N, Kurtoglu S. The effects of diet quality and dietary acid load on insulin resistance in overweight children and adolescents. Endocrinol Diabetes Nutr. 2021;69:426. 10.1016/j.endien.2022.06.001 35817547

[21] Cho J, Hong H, Park S, Kim S, Kang H. Insulin resistance and its association with metabolic syndrome in Korean children. Biomed Res Int. 2017;2017:8728017. 29457038 10.1155/2017/8728017 PMC5804402

[22] Kerr JA, Liu RS, Gasser CE, Mensah FK, Burgner D, Lycett K, et al. Diet quality trajectories and cardiovascular phenotypes/metabolic syndrome risk by 11–12 years. Int J Obes (Lond). 2021;45(7):1392–403. 33824404 10.1038/s41366-021-00800-x

[23] Rethlefsen ML, Kirtley S, Waffenschmidt S, Ayala AP, Moher D, Page MJ, et al. PRISMA-S: an extension to the PRISMA Statement for Reporting Literature Searches in Systematic Reviews. Syst Rev. 2021;10(1):39. 33499930 10.1186/s13643-020-01542-z PMC7839230

[24] Blaxter M. Criteria for the evaluation of qualitative research papers. Med Sociol News. 1996;22:68–71.

[25] Wells G, Shea B, O’Connell D, Peterson J. Ottawa, ON. Ottawa Hospital Research Institute. The Newcastle-Ottawa Scale (NOS) for assessing the quality of nonrandomised studies in meta-analyses. 2000. Available from: https://www.ohri.ca/programs/clinical_epidemiology/oxford.asp.

[26] NHLBI. Study Quality Assessment Tools | NHLBI, NIH. National Heart, Lung, and Blood Institute. Available from: https://www.nhlbi.nih.gov/health-topics/study-quality-assessment-tools.

[27] Higgins JPT, Thomas J, Chandler J, Cumpston M, Li T, Page M et al. Cochrane Handbook for Systematic Reviews of Interventions. England:Wiley. 2019. 10.1002/14651858.ED000142 PMC10284251 31643080

[28] Jiménez-Pavón D, Sesé MA, Huybrechts I, Cuenca-García M, Palacios G, Ruiz JR, et al. Dietary and lifestyle quality indices with/without physical activity and markers of insulin resistance in European adolescents: The HELENA study. Br J Nutr. 2013;110(10):1919–25. 23596986 10.1017/S0007114513001153

[29] Kanellopoulou A, Giannakopoulou SP, Notara V, Antonogeorgos G, Rojas-Gil AP, Kornilaki EN, et al. The association between adherence to the Mediterranean diet and childhood obesity; the role of family structure: Results from an epidemiological study in 1728 Greek students. Nutr Health. 2021;27(1):39–47. 33073650 10.1177/0260106020952600

[30] Katsagoni CN, Psarra G, Georgoulis M, Tambalis K, Panagiotakos DB, Sidossis LS. High and moderate adherence to Mediterranean lifestyle is inversely associated with overweight, general and abdominal obesity in children and adolescents: The MediLIFE-index. Nutr Res. 2020;73:38–47. 31841746 10.1016/j.nutres.2019.09.009

[31] Lakka TA, Lintu N, Väistö J, Viitasalo A, Sallinen T, Haapala EA, et al. A 2 year physical activity and dietary intervention attenuates the increase in insulin resistance in a general population of children: the PANIC study. Diabetologia. 2020;63(11):2270–81. 32816094 10.1007/s00125-020-05250-0 PMC7527318

[32] Linardakis M, Bertsias G, Sarri K, Papadaki A, Kafatos A. Metabolic syndrome in children and adolescents in Crete, Greece, and association with diet quality and physical fitness. J Public Health (Bangkok). 2008;16(6):421–8.

[33] Mistretta A, Marventano S, Antoci M, Cagnetti A, Giogianni G, Nolfo F, et al. Mediterranean diet adherence and body composition among Southern Italian adolescents. Obes Res Clin Pract. 2017;11(2):215–26. 27269367 10.1016/j.orcp.2016.05.007

[34] Murakami K, Livingstone MBE. Associations between energy density of meals and snacks and overall diet quality and adiposity measures in British children and adolescents: the National Diet and Nutrition Survey. Br J Nutr. 2016;116(9):1633–45. 27823581 10.1017/S0007114516003731

[35] Murakami K. Associations between nutritional quality of meals and snacks assessed by the Food Standards Agency nutrient profiling system and overall diet quality and adiposity measures in British children and adolescents. Nutrition. 2018;49(February 2018):57–65. 29499494 10.1016/j.nut.2017.10.011

[36] Perry CP, Keane E, Layte R, Fitzgerald AP, Perry IJ, Harrington JM. The use of a dietary quality score as a predictor of childhood overweight and obesity. BMC Publ Health. 2015;15(1):1–9. 10.1186/s12889-015-1907-y. 10.1186/s12889-015-1907-y PMC4477494 26100985

[37] Tognon G, Hebestreit A, Lanfer A, Moreno LA, Pala V, Siani A, et al. Mediterranean diet, overweight and body composition in children from eight European countries: Cross-sectional and prospective results from the IDEFICS study. Nutr Metab Cardiovasc Dis. 2014;24(2):205–13. 23870847 10.1016/j.numecd.2013.04.013

[38] Archero F, Ricotti R, Solito A, Carrera D, Civello F, Di Bella R, et al. Adherence to the mediterranean diet among school children and adolescents living in northern Italy and unhealthy food behaviors associated to overweight. Nutrients. 2018;10(9):1–13. 10.3390/nu10091322 PMC6165180 30231531

[39] Galan-Lopez P, Ries F, Gisladottir T, Domínguez R, Sánchez-Oliver AJ. Healthy lifestyle: Relationship between mediterranean diet, body composition and physical fitness in 13 to 16-years old icelandic students. Int J Environ Res Publ Health. 2018;15(12):2632. 10.3390/ijerph15122632 PMC6313697 30477217

[40] Alonso FJ, Carranza MD, Rueda JD, Naranjo J. Composición corporal en escolares de primaria y su relación con el habito nutricional y la práctica reglada de actividad física. Rev Andaluza Med del Deport. 2019;43(2):176.

[41] Calatayud Sáez F, Calatayud Moscoso del Prado B, Gallego Fernández-Pacheco JG. Efectos de una dieta mediterránea tradicional en niños con sobrepeso y obesidad tras un año de intervención. Pediatr Aten Primaria. 2011;13(52):553–69.

[42] Galan-Lopez P, Sánchez-Oliver AJ, Ries F, González-Jurado JA. Mediterranean diet, physical fitness and body composition in sevillian adolescents: A healthy lifestyle. Nutrients. 2019;11(9):2009. 31454923 10.3390/nu11092009 PMC6769614

[43] Notario-Barandiaran L, Valera-Gran D, Gonzalez-Palacios S, Garcia-de-la-Hera M, Fernández-Barrés S, Pereda-Pereda E, et al. High adherence to a mediterranean diet at age 4 reduces overweight, obesity and abdominal obesity incidence in children at the age of 8. Int J Obes. 2020;44(9):1906–17. 10.1038/s41366-020-0557-z 32152497

[44] Ojeda-Rodríguez A, Zazpe I, Morell-Azanza L, Chueca MJ, Azcona-Sanjulian MC, Marti A. Improved diet quality and nutrient adequacy in children and adolescents with abdominal obesity after a lifestyle intervention. Nutrients. 2018;10(10):1500. 30322156 10.3390/nu10101500 PMC6213517

[45] Rodríguez Cabrero M, García Aparicio A, Salinero JJ, Pérez González B, Sánchez Fernández JJ, Gracia R, et al. Calidad de la dieta y su relación con el IMC y el sexo en adolescentes. Nutr Clin Diet Hosp. 2012;32(2):21–7.

[46] Rosa Guillamón A, Carrillo López PJ, GarcíaCantó E, Perez Soto JJ, Tarraga Marcos L, Tarraga López PJ. Mediterranean diet, weight status and physical activity in schoolchildren of the Region of Murcia. Clín Investig Arterioscler (English Ed). 2019;31(1):1–7. 10.1016/j.arteri.2018.09.002 30503075

[47] Serrano MM, Camacho GO, Cardenosa MC, Cardenosa AC, Guardado IM, Sayavera JB, et al. Influencia de la calidad de la dieta de escolares sobre parametros sanguineus y antropométricos relacionados con la salud. Rev Esp Nutr Comunitaria. 2016;22(3):9–13.

[48] Nenadić DB, Kolak E, Selak M, Smoljo M, Radić J, Vučković M, et al. Anthropometric parameters and mediterranean diet adherence in preschool children in Split-Dalmatia county, Croatia—Are they related? Nutrients. 2021;13(12):4252. 34959811 10.3390/nu13124252 PMC8706144

[49] Bacopoulou F, Landis G, Rentoumis A, Tsitsika A, Efthymiou V. Mediterranean diet decreases adolescent waist circumference. Eur J Clin Invest. 2017;47(6):447–55. 28407234 10.1111/eci.12760

[50] Fernández-Iglesias R, Álvarez-Pereira S, Tardón A, Fernández-García B, Iglesias-Gutiérrez E. Adherence to the mediterranean diet in a school population in the principality of Asturias (Spain): Relationship with physical activity and body weight. Nutrients. 2021;13(5):1507. 33946967 10.3390/nu13051507 PMC8145401

[51] Gallardo LP, Adell CJ, Gonzalo DG, Díez JAR, Alcoceba RA. Weight status and adherence to the mediterranean diet in children from 6 to 9 years old in the interval of 10 years. Nutr Clin y Diet Hosp. 2021;41(2):53–60.

[52] George ES, Gavrili S, Itsiopoulos C, Manios Y, Moschonis G. Poor adherence to the Mediterranean diet is associated with increased likelihood of metabolic syndrome components in children: The Healthy Growth Study. Public Health Nutr. 2021;24(10):2823–33. 33866986 10.1017/S1368980021001701 PMC9884535

[53] Grams L, Nelius AK, Pastor GG, Sillero-Quintana M, Veiga ÓL, Homeyer D, et al. Comparison of adherence to mediterranean diet between Spanish and German school-children and influence of gender, overweight, and physical activity. Nutrients. 2022;14(21):4697. 36364959 10.3390/nu14214697 PMC9655044

[54] Martíncrespo-Blanco MC, Varillas-Delgado D, Blanco-Abril S, Cid-Exposito MG, Robledo-Martín J. Effectiveness of an Intervention Programme on Adherence to the Mediterranean Diet in a Preschool Child: A Randomised Controlled Trial. Nutrients. 2022;14(8). 10.3390/nu14081536 PMC9025428 35458098

[55] Seral-Cortes M, Sabroso-Lasa S, Bailo-Aysa A, Gonzalez-Gross M, Molnár D, Censi L, et al. Mediterranean diet, screen-time-based sedentary behavior and their interaction effect on adiposity in European adolescents: The HELENA study. Nutrients. 2021;13(2):1–16. 10.3390/nu13020474 PMC7911943 33573364

[56] Blijleven KA, Navis G, Kromhout D, Corpeleijn E. Nutrition beyond the first 1000 days: Diet quality and 7-year change in BMI and overweight in 3-year old children from the Dutch GECKO Drenthe birth cohort. J Dev Orig Health Dis. 2021;12(6):933–9. 33303050 10.1017/S204017442000118X

[57] Fernández-Álvarez MM, Martín-Payo R, Zabaleta-del-Olmo E, García-García R, Cuesta M, Gonzalez-Méndez X. Assessment of diet quality and physical activity of soccer players aged 13 to 16, from the principality of Asturias, Spain. An Pediatría (English Ed). 2021;95(1):33–9. 10.1016/j.anpede.2020.05.015. 10.1016/j.anpede.2020.05.015 34119459

[58] Farajian P, Risvas G, Karasouli K, Pounis GD, Kastorini CM, Panagiotakos DB, et al. Very high childhood obesity prevalence and low adherence rates to the Mediterranean diet in Greek children: the GRECO study. Atherosclerosis. 2011;217(2):525–30. 21561621 10.1016/j.atherosclerosis.2011.04.003

[59] Galan-Lopez P, Domínguez R, Pihu M, Gísladóttir T, Sánchez-Oliver AJ, Ries F. Evaluation of physical fitness, body composition, and adherence to mediterranean diet in adolescents from Estonia: The adoleshealth study. Int J Environ Res Public Health. 2019;16(22):1–13. 10.3390/ijerph16224479 PMC6888343 31739416

[60] De Santi M, Callari F, Brandi G, Toscano RV, Scarlata L, Amagliani G, et al. Mediterranean diet adherence and weight status among Sicilian Middle school adolescents. Int J Food Sci Nutr. 2020;71(8):1010–8. 32312138 10.1080/09637486.2020.1751089

[61] Er V, Dias KI, Papadaki A, White J, Wells S, Ward DS, et al. Association of diet in nurseries and physical activity with zBMI in 2-4-year olds in England: A cross-sectional study 11 medical and health sciences 1117 public health and health services. BMC Public Health. 2018;18(1):1–11. 10.1186/s12889-018-6138-6 PMC6236905 30428858

[62] Galan-Lopez P, Gisladóttir T, Ries F. Adherencia a la Dieta Mediterránea, Motivos para la Práctica de Ejercicio Físico y Composición Corporal en Adolescentes Islandeses (Adherence to the Mediterranean Diet, Motives for Physical Exercise and Body Composition in Icelandic Adolescents). Retos. 2020;2041(38):552–9.

[63] Galan-Lopez P, Sanchez-Oliver AJ, Pihu M, Gísladóttír T, Domínguez R, Ries F. Association between adherence to the mediterranean diet and physical fitness with body composition parameters in 1717 european adolescents: The adoleshealth study. Nutrients. 2020;12(1):1–19. 10.3390/nu12010077 PMC7019378 31892139

[64] Asghari G, Yuzbashian E, Mirmiran P, Hooshmand F, Najafi R, Azizi F. Dietary approaches to stop hypertension (DASH) dietary pattern is associated with reduced incidence of metabolic syndrome in children and adolescents. J Pediatr. 2016;174:178-184.e1. 10.1016/j.jpeds.2016.03.077. 27156186 10.1016/j.jpeds.2016.03.077

[65] Golpour-hamedani S, Mohammadifard N, Khosravi A. Dietary approaches to stop hypertension diet and obesity: A cross-sectional study of Iranian children and adolescents. ARYA Atheroscler. 2017;13(1):7–13. 28761449 PMC5515185

[66] Hooshmand F, Asghari G, Yuzbashian E, Mahdavi M, Mirmiran P, Azizi F. Modified healthy eating index and incidence of metabolic syndrome in children and adolescents: Tehran lipid and glucose study. J Pediatr. 2018;197(April):134-139.e2. 29631767 10.1016/j.jpeds.2018.01.080

[67] Korkmaz GÖ, Kabaran S. Protective effects of a Mediterranean-like dietary pattern on obesity, abdominal obesity and large neck circumference in a cohort of Turkish children aged 6–9 years. Asia Pac J Clin Nutr. 2020;29(2):363–71. 32674244 10.6133/apjcn.202007_29(2).0019

[68] Mohseni-Takalloo S, Hosseini-Esfahani F, Mirmiran P, Azizi F. Associations of pre-defined dietary patterns with obesity associated phenotypes in Tehranian adolescents. Nutrients. 2016;8(8):505. 27548211 10.3390/nu8080505 PMC4997418

[69] Sümen A, Evgin D. A cross-sectional study examining self-reported anthropometric measurements with adolescents’ nutrition attitudes, obesity awareness and diet quality indices during the pandemic. J Pediatr Nurs. 2022;64:133. 35181175 10.1016/j.pedn.2022.01.018 PMC9759513

[70] Çağiran Yilmaz F, Çağiran D, Özçelik AÖ. Adolescent obesity and its association with diet quality and cardiovascular risk factors. Ecol Food Nutr. 2019;58(3):207–18. 30786756 10.1080/03670244.2019.1580581

[71] Asgari E, Chamary M, Bellissimo N, Azadbakht L. Association between adherence to the MIND diet and overweight and obesity in children: An exploratory study. Clin Nutr ESPEN. 2022;51:313–8. 10.1016/j.clnesp.2022.08.008. 36184222 10.1016/j.clnesp.2022.08.008

[72] Askari M, Daneshzad E, Naghshi S, Bellissimo N, Suitor K, Azadbakht L. Healthy eating index and anthropometric status in young children: A cross-sectional study. Clin Nutr ESPEN. 2021;45(xxxx):306–11. 10.1016/j.clnesp.2021.07.030. 34620333 10.1016/j.clnesp.2021.07.030

[73] Kocaadam-Bozkurt B, Karaçil Ermumcu MŞ, Erdoğan Gövez N, Bozkurt O, Akpinar Ş, Mengi Çelik Ö, et al. Association between adherence to the Mediterranean diet with anthropometric measurements and nutritional status in adolescents. Nutr Hosp. 2023;40(2):368–76. 36880734 10.20960/nh.04545

[74] Lioret S, McNaughton SA, Cameron AJ, Crawford D, Campbell KJ, Cleland VJ, et al. Three-year change in diet quality and associated changes in BMI among schoolchildren living in socio-economically disadvantaged neighbourhoods. Br J Nutr. 2014;112(2):260–8. 24775601 10.1017/S0007114514000749 PMC5385210

[75] Wong JE, Parnell WR, Howe AS, Lubransky AC, Black KE, Skidmore PML. Diet quality is associated with measures of body fat in adolescents from Otago. New Zealand Public Health Nutr. 2015;18(8):1453–60. 25158609 10.1017/S1368980014001645 PMC10271662

[76] Sahel K, Elfane H, El-Jamal S, El Ayachi M, Belahsen R. Food quality and nutritional status of school-going adolescents in the province of El Jadida in Morocco. Rocz Panstw Zakl Hig / Ann Natl Inst Hyg. 2022;73(4):423–33. 10.32394/rpzh.2022.0237 36546881

[77] An R. Diet quality and physical activity in relation to childhood obesity. Int J Adolesc Med Health. 2017; 29(2). 10.1515/ijamh-2015-0045 26351906

[78] Summer SS, Jenkins T, Inge T, Deka R, Khoury JC. Association of diet quality, physical activity, and abdominal obesity with metabolic syndrome z-score in black and white adolescents in the US. Nutr Metab Cardiovasc Dis. 2022;32(2):346–54. 34953632 10.1016/j.numecd.2021.10.021 PMC8802754

[79] Liu M, Chen Q, Li Z, Zhang J, Wang P, He Q. Association between diet quality and cardiometabolic risk factor clustering stratified by socioeconomic status among chinese children. J Acad Nutr Diet. 2021;121(10):1975–19832. 33893062 10.1016/j.jand.2021.03.009

[80] Hu K, Button AM, Tate CM, Kracht CL, Champagne CM, Staiano AE. Adolescent diet quality, cardiometabolic risk, and adiposity: A prospective cohort. J Nutr Educ Behav. 2023;55(12):851–60. 37897452 10.1016/j.jneb.2023.10.003 PMC10842960

[81] Zheng X, Wang H, Wu H. Association between diet quality scores and risk of overweight and obesity in children and adolescents. BMC Pediatr. 2023;23(1):1–8. 37046233 10.1186/s12887-023-03966-7 PMC10100112

[82] Berz JPB, Singer MR, Guo X, Daniels SR, Moore LL. Use of a DASH food group score to predict excess weight gain in adolescent girls in the national growth and health study. Arch Pediatr Adolesc Med. 2011;165:540–6. 21646587 10.1001/archpediatrics.2011.71

[83] Thomson JL, Landry AS, Tussing-Humphreys LM, Goodman MH. Diet quality of children in the United States by body mass index and sociodemographic characteristics. Obes Sci Pract. 2020;6(1):84–98. 32128246 10.1002/osp4.388 PMC7042025

[84] Hajna S, Liu J, Leblanc PJ, Faught BE, Merchant AT, Cairney J, et al. Association between body composition and conformity to the recommendations of Canada’s Food Guide and the Dietary Approaches to Stop Hypertension (DASH) diet in peri-adolescence. Public Health Nutr. 2012;15(10):1890–6. 22717343 10.1017/S1368980012001024

[85] McGee M, Unger S, Hamilton J, Birken CS, Pausova Z, Kiss A, et al. Associations between diet quality and body composition in young children born with very low body weight. J Nutr. 2020;150(11):2961–8. 33025010 10.1093/jn/nxaa281 PMC7675023

[86] Setayeshgar S, Maximova K, Ekwaru JP, Gray-Donald K, Henderson M, Paradis G, et al. Diet quality as measured by the Diet Quality Index-International is associated with prospective changes in body fat among Canadian children. Public Health Nutr. 2017;20(3):456–63. 27660199 10.1017/S1368980016002500 PMC10261485

[87] Bekelman TA, Ringham BM, Sauder KA, Johnson SL, Harrall KH, Glueck DH, et al. Adherence to index-based dietary patterns in childhood and BMI trajectory during the transition to adolescence: the EPOCH study. Int J Obes. 2022;45(11):2439–46. 10.1038/s41366-021-00917-z PMC8542564 34304241

[88] Ducharme-Smith K, Brady TM, Vizthum D, Caulfield LE, Mueller NT, Rosenstock S, et al. Diet quality scores associated with improved cardiometabolic measures among African American adolescents. Pediatr Res. 2022;92(3):853–61. 34916627 10.1038/s41390-021-01893-w PMC8674518

[89] Ducharme-Smith K, Caulfield LE, Brady TM, Rosenstock S, Mueller NT, Garcia-Larsen V. Higher Diet Quality in African-American Adolescents Is Associated with Lower Odds of Metabolic Syndrome: Evidence from the NHANES. J Nutr. 2021;151(6):1609–17. 33768240 10.1093/jn/nxab027 PMC9034326

[90] Torres R, Santos E, Orraca L, Elias A, Palacios C. Diet quality, social determinants, and weight status in puerto rican children aged 12 years. J Acad Nutr Diet. 2014;114(8):1230–5. 24656710 10.1016/j.jand.2014.01.011 PMC4111983

[91] Torres R, Serrano M, Pérez CM, Palacios C. Physical environment, diet quality, and body weight in a group of 12-year-old children from four public schools in Puerto Rico. P R Health Sci J. 2014;33(1):14–21. 24665604 PMC4142497

[92] Aljahdali AA, Peterson KE, Cantoral A, Ruiz-Narvaez E, Tellez-Rojo MM, Kim HM, et al. Diet quality scores and cardiometabolic risk factors in Mexican children and adolescents: A longitudinal analysis. Nutrients. 2022;14(4):1–19. 10.3390/nu14040896 PMC8878155 35215546

[93] Bricarello LP, de Almeida Alves M, Retondario A, de Moura Souza A, de Vasconcelos AG. DASH diet (Dietary Approaches to Stop Hypertension) and overweight/obesity in adolescents: The ERICA study. Clin Nutr ESPEN. 2021;42:173–9. 10.1016/j.clnesp.2021.02.001. 33745574 10.1016/j.clnesp.2021.02.001

[94] Latorre-Román PÁ, Guzmán-Guzmán IP, Antonio Párraga-Montilla J, Caamaño-Navarrete F, Salas-Sánchez J, Palomino-Devia C, et al. Healthy lifestyles and physical fitness are associated with abdominal obesity among Latin-American and Spanish preschool children: A cross-cultural study. Pediatr Obes. 2022;17(7):1–8. 10.1111/ijpo.12901 PMC9286564 35233958

[95] Pereira JL, Mattei J, Isasi CR, Van Horn L, Carnethon MR, Daviglus ML, et al. Diet quality, excess body weight and cardiometabolic risk factors in adolescents living in São Paulo, Brazil and in the USA: Differences and similarities. Public Health Nutr. 2021;24(13):4091–101. 32907665 10.1017/S1368980020002736 PMC8501489

[96] Said FA, Khamis AG, Salmin AH, Msellem SN, Mdachi K, Noor R, et al. Influence of diet quality on nutritional status of school-aged children and adolescents in Zanzibar, Tanzania. PLoS ONE. 2023;18(10):1–12. 10.1371/journal.pone.0293316 PMC10588882 37862346

[97] Sørensen LMN, Aamodt G, Brantsæter AL, Meltzer HM, Papadopoulou E. Diet quality of Norwegian children at 3 and 7 years: changes, predictors and longitudinal association with weight. Int J Obes. 2022;46(1):10–20. 10.1038/s41366-021-00951-x. 10.1038/s41366-021-00951-x 34462565

[98] Rolland-Cachera MF, Deheeger M, Maillot M, Bellisle F. Early adiposity rebound: causes and consequences for obesity in children and adults. Int J Obes. 2006;30(4):S11-7. 10.1038/sj.ijo.0803514 17133230

[99] D’innocenzo S, Biagi C, Lanari M. Obesity and the mediterranean diet: A review of evidence of the role and sustainability of the mediterranean diet. Nutrients. 2019;11(6):1306. 31181836 10.3390/nu11061306 PMC6627690

[100] Tognon G, Moreno LA, Mouratidou T, Veidebaum T, Molnár D, Russo P, et al. Adherence to a Mediterranean-like dietary pattern in children from eight European countries. The IDEFICS study. Int J Obes. 2014;38:S108–14. 10.1038/ijo.2014.141 25219407

[101] Castro-Barquero S, Ruiz-León AM, Sierra-Pérez M, Estruch R, Casas R. Dietary strategies for metabolic syndrome: A comprehensive review. Nutrients. 2020;12(10):1–21. 10.3390/nu12102983 PMC7600579 33003472

[102] Schap TR, Kuczynski K, Hiza H. Healthy eating index—beyond the score. J Acad Nutr Diet. 2017;117(4):519–21. 28343522 10.1016/j.jand.2017.02.002

[103] Kim S, Haines PS, Siega-Riz AM, Popkin BM. The diet quality index-international (DQI-I) provides an effective tool for cross-national comparison of diet quality as illustrated by China and the United States. J Nutr. 2003;133(11):3476–84. 14608061 10.1093/jn/133.11.3476

[104] Hlaing-hlaing H, Pezdirc K, Tavener M, James EL, Hure A. Diet quality indices used in Australian and New Zealand Adults: A systematic review and critical appraisal. Nutrients. 2020;12(12):1–30. 10.3390/nu12123777 PMC7763901 33317123

[105] Yoong SL, Skelton E, Jones J, Wolfenden L. Do childcare services provide foods in line with the 2013 Australian Dietary guidelines? A cross-sectional study. Aust N Z J Public Health. 2014;38(6):595–6. 25440467 10.1111/1753-6405.12312

[106] Costarelli V, Michou M, Panagiotakos DB, Lionis C. Adherence to the Mediterranean diet and weight status in children: the role of parental feeding practices. Int J Food Sci Nutr. 2021;72(1):112–22. 32458711 10.1080/09637486.2020.1765151

