# Supplementary material for: Diet Quality Scores, Obesity and Metabolic Syndrome in Children and Adolescents: A Systematic Review and Meta-Analysis
Source: Curr Obes Rep. 2024 Sep 27;13(4):755–88. doi: 10.1007/s13679-024-00589-6 (PMC11522196; doi:10.1007/s13679-024-00589-6)
Supplement: Supplementary file 1 — Supplementary file1 (DOCX 35 KB) [file 13679_2024_589_MOESM1_ESM.docx]

Diet quality scores, obesity and metabolic syndrome in children and adolescents: A systematic review and meta-analysis.

Current Obesity Reports

Alicia Larruy-García^1^, Lubna Mahmood^1^, María L. Miguel-Berges^1,2^, Guiomar Masip^1^, Miguel Seral-Cortés^1,2^, Pilar De Miguel-Etayo^1,2^, Luis A. Moreno^1,2^

^1^ Growth, Exercise, Nutrition and Development (GENUD) Research Group, Instituto Agroalimentario de Aragón (IA2). Physiatry and Nursing Department, Faculty of Health Sciences. Universidad de Zaragoza, Instituto de Investigación Sanitaria de Aragón (IIS Aragón), 50009 Zaragoza, Spain.

^2^ CIBER. Fisiopatología de la Obesidad y Nutrición (CIBEROBN), Instituto de Salud Carlos III (ISCIII), Madrid, Spain.

Corresponding author: Pilar De Miguel-Etayo.

Contact address: Growth, Exercise, NUtrition and Development (GENUD) Research Group, Faculty of Health Sciences, Pedro Cerbuna Street, Universidad de Zaragoza, 50009 Zaragoza, Spain

E-mail address: [pilardm@unizar.es](mailto:pilardm@unizar.es)

Alicia Larruy-García ORCID ID: [0000-0002-2165-5263](https://orcid.org/0000-0002-2165-5263)

Lubna Mahmood ORCID ID: N/A

María L Miguel-Berges ORCID ID: [0000-0002-2411-9538](https://orcid.org/0000-0002-2411-9538)

Guiomar Masip ORCID ID:  [0000-0001-6311-5276](http://orcid.org/0000-0001-6311-5276)

Miguel Seral-Cortés ORCID ID: [0000-0003-2198-2704](https://orcid.org/0000-0003-2198-2704)

Pilar De Miguel-Etayo ORCID ID: [0000-0001-6173-5850](https://orcid.org/0000-0001-6173-5850)

Luis A Moreno ORCID ID: [0000-0003-0454-653X](https://orcid.org/0000-0003-0454-653X)

Summary of the Quality Assessment Tools

| **Study Design** | **Quality Assessment Tool** | **Quality Ranking of study** |
| --- | --- | --- |
| Cross-sectional study | BSA Medical Sociology Group | Score 1-7 |
|  |  | 1-2 (Low) |
|  |  | 3-5 (Moderate) |
|  |  | 6-7 (High) |
| Cohort study | Quality assessment scale of Newcastle-Ottawa | Score 0-9 |
|  |  | 0-3 (Low) |
|  |  | 4-6 (Medium) |
|  |  | 7-9 (High) |
| Interventional study | The National Heart, Lung, and Blood Institute quality assessment of controlled intervention studies tool | Score 0-14 |
|  |  | 0-5 (Low) |
|  |  | 6-9 (Medium) |
|  |  | 10-14 (High) |

**Table Q1** Quality Control Assessment of included studies

| **Author** | **Year of Publication** | **Study Design** | **Quality assessment tool** | **Quality Rating of study** | **Comments** |
| --- | --- | --- | --- | --- | --- |
| Ali Said F et al | 2023 | Cross-sectional study | BSA Medical Sociology Group | 6 | High |
| Aljahdali AA et al | 2022 | Cohort study | Quality assessment scale of Newcastle-Ottawa | 8 | High |
| Alonso FJ et al | 2014 | Cohort study | Quality assessment scale of Newcastle-Ottawa | 7 | High |
| Archero F et al | 2018 | Cross-sectional study | BSA Medical Sociology Group | 5 | Moderate |
| Asgari E et al | 2022 | Cross-sectional study | BSA Medical Sociology Group | 5 | Moderate |
| Asghari G et al | 2016 | Cross-sectional study | BSA Medical Sociology Group | 6 | High |
| Askari M et al | 2021 | Cross-sectional study | BSA Medical Sociology Group | 5 | Moderate |
| Bacopoulou F et al | 2017 | Cross-sectional study | BSA Medical Sociology Group | 5 | Moderate |
| Bekelman TA et al | 2021 | Cohort study | Quality assessment scale of Newcastle-Ottawa | 7 | High |
| Berz JPB et al | 2011 | Cohort study | Quality assessment scale of Newcastle-Ottawa | 8 | High |
| Bricarello LP et al | 2021 | Cross-sectional study | BSA Medical Sociology Group | 6 | High |
| Bučan Nenadić D et al | 2021 | Cross-sectional study | BSA Medical Sociology Group | 5 | Moderate |
| Calatayud Saez F et al | 2011 | Cohort study | Quality assessment scale of Newcastle-Ottawa | 7 | High |
| De Miguel Etayo P et al | 2019 | Cohort study | Quality assessment scale of Newcastle-Ottawa | 8 | High |
| De Santi M et al | 2020 | Cross-sectional study | BSA Medical Sociology Group | 5 | Moderate |
| Ducharme-Smith K et al | 2021 | Cross-sectional study | BSA Medical Sociology Group | 6 | High |
| Ducharme-Smith K et al | 2021 | Cross-sectional study | BSA Medical Sociology Group | 4 | Moderate |
| Er V et al | 2018 | Cross-sectional study | BSA Medical Sociology Group | 6 | High |
| Farajian P et al | 2011 | Cross-sectional study | BSA Medical Sociology Group | 7 | High |
| Fernández-Álvarez MDM et al | 2021 | Cross-sectional study | BSA Medical Sociology Group | 6 | High |
| Fernández-Iglesias R et al | 2021 | Cross-sectional study | BSA Medical Sociology Group | 7 | High |
| Galan-López P et al | 2018 | Cross-sectional study | BSA Medical Sociology Group | 6 | High |
| Galan-López P et al | 2019 | Cross-sectional study | BSA Medical Sociology Group | 5 | Moderate |
| Galan-López P et al | 2019 | Cross-sectional study | BSA Medical Sociology Group | 5 | Moderate |
| Galan-López P et al | 2019 | Cross-sectional study | BSA Medical Sociology Group | 6 | High |
| Galan-López P et al | 2020 | Cross-sectional study | BSA Medical Sociology Group | 6 | High |
| Gallardo LP et al | 2021 | Descriptive transversal | ND | ND | ND |
| George ES et al | 2021 | Cross-sectional study | BSA Medical Sociology Group | 7 | High |
| Golpour-Hamedani S et al | 2017 | Cross-sectional study | BSA Medical Sociology Group | 6 | High |
| Grams L et al | 2022 | Cohort study | Quality assessment scale of Newcastle-Ottawa | 7 | High |
| Hanja S et al | 2012 | Cross-sectional study | BSA Medical Sociology Group | 5 | Moderate |
| Hooshmand F et al | 2018 | Cohort study | Quality assessment scale of Newcastle-Ottawa | 8 | High |
| Hu K et al | 2023 | Cross-sectional study | BSA Medical Sociology Group | 6 | High |
| Jiménez-Pavón D et al | 2013 | Cross-sectional study | BSA Medical Sociology Group | 5 | Moderate |
| Kanellopoulou A et al | 2020 | Cross-sectional study | BSA Medical Sociology Group | 7 | High |
| Katsagoni CN et al | 2019 | Cross-sectional study | BSA Medical Sociology Group | 5 | Moderate |
| Kocaadam-Bozkurt B et al | 2023 | Cross-sectional study | BSA Medical Sociology Group | 5 | Moderate |
| Korkmaz GO et al | 2020 | Cross-sectional study | BSA Medical Sociology Group | 5 | Moderate |
| Lakka TA et al | 2020 | Interventional study | The National Heart, Lung, and Blood Institute | 9 | Moderate |
| Latorre-Roman P et al | 2022 | Cross-sectional study | BSA Medical Sociology Group | 5 | Moderate |
| Linardakis M et al | 2008 | Cross-sectional study | BSA Medical Sociology Group | 6 | High |
| Lioret S et al | 2014 | Cohort study | Quality assessment scale of Newcastle-Ottawa | 8 | High |
| Liu M et al | 2021 | Cohort study | Quality assessment scale of Newcastle-Ottawa | 8 | High |
| Marcos-Serrano M et al | 2016 | Cross-sectional study | BSA Medical Sociology Group | 5 | Moderate |
| Martincrespo-Blanco MC et al | 2022 | Interventional study | The National Heart, Lung, and Blood Institute | 9 | Medium |
| McGee M et al | 2020 | Cohort study | Quality assessment scale of Newcastle-Ottawa | 8 | High |
| Mistretta A et al | 2020 | Cross-sectional study | BSA Medical Sociology Group | 6 | High |
| Mohseni-Takalloo S et al | 2016 | Cross-sectional study | BSA Medical Sociology Group | 5 | Moderate |
| Munrakami K et al | 2016 | Cross-sectional study | BSA Medical Sociology Group | 6 | High |
| Munrakami K et al | 2018 | Cross-sectional study | BSA Medical Sociology Group | 6 | High |
| Notario-Barandian L et al | 2020 | Cross-sectional study | BSA Medical Sociology Group | 5 | Moderate |
| Ojeda-Rodriguez A et al | 2018 | Interventional study | The National Heart, Lung, and Blood Institute | 9 | Medium |
| Pan Y et al | 2008 | Cross-sectional study | BSA Medical Sociology Group | 5 | Moderate |
| Pereira JL et al | 2021 | Cross-sectional study | BSA Medical Sociology Group | 4 | Moderate |
| Perry CP et al | 2005 | Cohort study | Quality assessment scale of Newcastle-Ottawa | 8 | High |
| Rodriguez-Cabrero M et al | 2012 | Cross-sectional study | BSA Medical Sociology Group | 4 | Moderate |
| Rosa Guillamón A et al | 2019 | Cross-sectional study | BSA Medical Sociology Group | 5 | Moderate |
| Ruopeng An et al | 2015 | Cross-sectional study | BSA Medical Sociology Group | 5 | Moderate |
| Sá Lustosa LCR et al | 2019 | Cross-sectional study | BSA Medical Sociology Group | 5 | Moderate |
| Sahel K et al | 2022 | Cross-sectional study | BSA Medical Sociology Group | 4 | Moderate |
| Seral-Cortés M et al | 2021 | Cross-sectional study | BSA Medical Sociology Group | 5 | Moderate |
| Setayeshgar S et al | 2016 | Cohort study | Quality assessment scale of Newcastle-Ottawa | 8 | High |
| Sørensen LMN et al | 2020 | Cohort study | Quality assessment scale of Newcastle-Ottawa | 8 | High |
| Sümen A et al | 2022 | Cross-sectional study | BSA Medical Sociology Group | 6 | Moderate |
| Summer SS et al | 2021 | Cross-sectional study | BSA Medical Sociology Group | 5 | Moderate |
| Thomson JL et al | 2019 | Cross-sectional study | BSA Medical Sociology Group | 5 | Moderate |
| Tognon G et al | 2013 | Interventional study | The National Heart, Lung, and Blood Institute | 7 | High |
| Torres R et al | 2014 | Cross-sectional study | BSA Medical Sociology Group | 5 | Moderate |
| Torres R et al | 2014 | Cross-sectional study | BSA Medical Sociology Group | 5 | Moderate |
| Vinke PC et al | 2020 | Cohort study | Quality assessment scale of Newcastle-Ottawa | 8 | High |
| Wong JE et al | 2014 | Cross-sectional study | BSA Medical Sociology Group | 6 | High |
| Yılmaz FÇ et al | 2021 | Cross-sectional study | BSA Medical Sociology Group | 5 | Moderate |
| Zheng X et al | 2023 | Cross-sectional study | BSA Medical Sociology Group | 5 | Moderate |
