# Supplementary material for: Diet Quality Scores, Obesity and Metabolic Syndrome in Children and Adolescents: A Systematic Review and Meta-Analysis
Source: Curr Obes Rep. 2024 Sep 27;13(4):755–88. doi: 10.1007/s13679-024-00589-6 (PMC11522196; doi:10.1007/s13679-024-00589-6)
Supplement: Supplementary file 2 — Supplementary file2 (DOCX 18 KB) [file 13679_2024_589_MOESM2_ESM.docx]

Diet quality scores, obesity and metabolic syndrome in children and adolescents: A systematic review and meta-analysis.

Current Obesity Reports

Alicia Larruy-García^1^, Lubna Mahmood^1^, María L. Miguel-Berges^1,2^, Guiomar Masip^1^, Miguel Seral-Cortés^1,2^, Pilar De Miguel-Etayo^1,2^, Luis A. Moreno^1,2^

^1^ Growth, Exercise, Nutrition and Development (GENUD) Research Group, Instituto Agroalimentario de Aragón (IA2). Physiatry and Nursing Department, Faculty of Health Sciences. Universidad de Zaragoza, Instituto de Investigación Sanitaria de Aragón (IIS Aragón), 50009 Zaragoza, Spain.

^2^ CIBER. Fisiopatología de la Obesidad y Nutrición (CIBEROBN), Instituto de Salud Carlos III (ISCIII), Madrid, Spain.

Corresponding author: Pilar De Miguel-Etayo.

Contact address: Growth, Exercise, NUtrition and Development (GENUD) Research Group, Faculty of Health Sciences, Pedro Cerbuna Street, Universidad de Zaragoza, 50009 Zaragoza, Spain

E-mail address: [pilardm@unizar.es](mailto:pilardm@unizar.es)

Alicia Larruy-García ORCID ID: [0000-0002-2165-5263](https://orcid.org/0000-0002-2165-5263)

Lubna Mahmood ORCID ID: N/A

María L Miguel-Berges ORCID ID: [0000-0002-2411-9538](https://orcid.org/0000-0002-2411-9538)

Guiomar Masip ORCID ID:  [0000-0001-6311-5276](http://orcid.org/0000-0001-6311-5276)

Miguel Seral-Cortés ORCID ID: [0000-0003-2198-2704](https://orcid.org/0000-0003-2198-2704)

Pilar De Miguel-Etayo ORCID ID: [0000-0001-6173-5850](https://orcid.org/0000-0001-6173-5850)

Luis A Moreno ORCID ID: [0000-0003-0454-653X](https://orcid.org/0000-0003-0454-653X)

**Table S1** Search strategy for systematic reviews and systematic review protocols

| **Database** | **Search Query** |
| --- | --- |
| **PubMed**  N= 878 | ("diet quality" OR "diet patterns" OR "diet score" OR “Healthy Eating Indices” OR "diet (quality) index" OR "diet indices" OR “Dietary Approaches To Stop Hypertension"[Mesh] OR “Dietary Approaches To Stop Hypertension” OR “DASH Diet” OR “Mediterranean Diet”) AND ("Metabolic Syndrome"[Title/Abstract] OR "Metabolic Syndrome"[MeSH Terms] OR "Insulin Resistance"[Title/Abstract] OR "Insulin Resistance"[MeSH Terms] OR "insulin sensitive"[Title/Abstract] OR “Obesity”[Title/Abstract] OR “Obesity”[MeSH Terms] OR “Pediatric Obesity”[Title/Abstract] OR “Pediatric Obesity”[MeSH Terms]) AND ("Body Composition"[Title/Abstract] OR "Body Composition"[MeSH Terms] OR “skinfold Thickness”[Title/Abstract] OR “Skinfold Thickness”[MeSH Terms] OR “Body fat distribution”[Title/Abstract] OR “Body fat distribution”[MeSH Terms] OR “Adiposity”[Title/Abstract] OR “Adiposity”[MeSH Terms] OR “Body weight”[Title/Abstract] OR “Body weight”[MeSH Terms] OR “Body weight and measures”[Title/Abstract] OR “Body Mass Index”[Title/Abstract] OR “Body Mass Index”[MeSH Terms] OR “Body fat distribution”[Title/Abstract] OR “Body fat distribution”[MeSH Terms] OR “Fat mass index”[Title/Abstract] OR “Quetelet Index”[Title/Abstract] OR “Waist circumference”[Title/Abstract] OR “Waist circumference”[MeSH Terms] OR “Waist-hip ratio”[Title/Abstract] OR “Waist-hip ratio”[MeSH Terms] OR “Waist-height ratio”[Title/Abstract] OR “Waist-height ratio”[MeSH Terms]) AND ("child*"[Title/Abstract] OR "child"[MeSH Terms] OR “Preschool*”[Title/Abstract] OR “Child, Preschool*”[MeSH Terms] OR "adolescen*"[Title/Abstract] OR "adolescent"[MeSH Terms] OR “Youth”[Title/Abstract] OR “Teen*”[Title/Abstract] OR “Young people”[Title/Abstract]) AND (humans[Filter]) |
| **Cochrane Library**  N= 169 | ("dietary quality" OR "dietary patterns" OR "diet score" OR “Healthy Eating Indices” OR "diet quality index" OR "diet indices" OR “Dietary Approaches To Stop Hypertension” OR “DASH Diet” OR “Mediterranean Diet”) AND (Child* OR Preschool* OR Adolescen* OR “Youth” OR Teen* OR “Young people”) AND ("Metabolic Syndrome" OR "Insulin Resistance" OR “Insulin sensitive” OR “Obesity” OR “Pediatric Obesity”) AND (“Body composition” OR “skinfolds” OR “Body fat distribution” OR “Adiposity” OR “Body weight” OR “Body weight and measures” OR “Body Mass Index” OR “Body fat” OR “Fat mass index” OR “Quetelet Index” OR “Waist circumference” OR “Waist-hip ratio” OR “Waist-height ratio”) in Title Abstract Keyword |
| **Embase**  N= 1110 | (‘diet quality’:ti,ab,kw OR ‘diet quality’/exp OR ‘dietary pattern’:ti,ab,kw OR ‘dietary pattern’/exp OR ‘diet score’:ti,ab,kw OR ‘Healthy Eating Index’:ti,ab,kw OR ‘Healthy Eating Index’/exp OR ‘diet quality index’:ti,ab,kw OR ‘diet quality index’/exp OR ‘diet indices’:ti,ab,kw OR ‘Dietary Approaches To Stop Hypertension’:ti,ab,kw OR ‘DASH Diet’:ti,ab,kw OR ‘DASH Diet’/exp OR ‘Mediterranean diet’:ti,ab,kw OR ‘Mediterranean diet’/exp) AND ('metabolic syndrome':ti,ab,kw OR 'metabolic syndrome x':ti,ab,kw OR 'metabolic syndrome x'/exp OR 'insulin sensitive':ti,ab,kw OR 'insulin sensitivity':ti,ab,kw OR 'insulin sensitivity'/exp OR 'insulin resistance':ti,ab,kw OR 'obesity':ti,ab,kw OR 'obesity'/exp OR 'childhood obesity':ti,ab,kw OR 'childhood obesity'/exp) AND ('body composition':ti,ab,kw OR 'body composition'/exp OR 'skinfold':ti,ab,kw OR 'skinfold'/exp OR 'body fat distribution':ti,ab,kw OR 'body fat distribution'/exp OR 'adiposity':ti,ab,kw OR 'body weight':ti,ab,kw OR 'body weight'/exp OR 'body weight and measures':ti,ab,kw OR 'body mass':ti,ab,kw OR 'body mass'/exp OR 'body fat':ti,ab,kw OR 'body fat'/exp OR 'fat mass index':ti,ab,kw OR 'quetelet index':ti,ab,kw OR 'waist circumference':ti,ab,kw OR 'waist circumference'/exp OR 'waist hip ratio':ti,ab,kw OR 'waist hip ratio'/exp OR 'waist to height ratio':ti,ab,kw OR 'waist to height ratio'/exp) AND ('child':ti,ab,kw OR 'child'/exp OR 'adolescen*':ti,ab,kw OR 'adolescence'/exp OR 'adolescent'/exp OR 'preschool':ti,ab,kw OR 'preschool'/exp OR 'juvenile':ti,ab,kw OR 'juvenile'/exp) AND [humans]/lim AND ([english]/lim OR [spanish]/lim) |
| **Scopus**  N= 1323 | TITLE-ABS-KEY(("dietary quality" OR "dietary patterns" OR "diet score" OR "Healthy Eating Indices" OR "diet quality index" OR "diet indices" OR "Dietary Approaches To Stop Hypertension" OR "DASH Diet" OR "Mediterranean Diet") AND (Child* OR Preschool* OR Adolescen* OR "Youth" OR Teen* OR "Young people") AND ("Metabolic Syndrome" OR "Insulin Resistance" OR "Insulin sensitive" OR "Obesity" OR "Pediatric Obesity") AND ("Body composition" OR "skinfolds" OR "Body fat distribution" OR "Adiposity" OR "Body weight" OR "Body weight and measures" OR "Body Mass Index" OR "Body fat" OR "Fat mass index" OR "Quetelet Index" OR "Waist circumference" OR "Waist-hip ratio" OR "Waist-height ratio")) AND ( LIMIT-TO ( PUBSTAGE,"final" ) ) AND ( LIMIT-TO ( DOCTYPE,"ar" ) ) AND ( LIMIT-TO ( LANGUAGE,"English" ) OR LIMIT-TO ( LANGUAGE,"Spanish" ) ) |
| **Scielo**  N= 39 | ((diet(ary) quality) OR (diet(ary) patterns) OR (diet score) OR (Healthy Eating Indices) OR (diet (quality) index) OR (diet indices) OR (Dietary Approaches To Stop Hypertension) OR (Dietary Approaches To Stop Hypertension) OR (DASH Diet) OR (Mediterranean Diet)) AND (Child* OR Preschool* OR Adolescen* OR (Youth) OR Teen* OR (Young people)) AND ((Metabolic Syndrome) OR (Insulin Resistance) OR (Insulin sensitive) OR (Obesity) OR (Pediatric Obesity)) AND ((Body composition) OR (skinfolds) OR (Body fat distribution) OR (Adiposity) OR (Body weight) OR (Body weight and measures) OR (Body Mass Index) OR (Body fat) OR (Fat mass index) OR (Quetelet Index) OR (Waist circumference) OR (Waist-hip ratio) OR (Waist-height ratio)) |
